# Robust induction of interferon and interferon-stimulated gene expression by influenza B/Yamagata lineage virus infection of A549 cells

**DOI:** 10.1371/journal.pone.0231039

**Published:** 2020-04-08

**Authors:** Pengtao Jiao, Wenhui Fan, Ying Cao, He Zhang, Lu Tian, Lei Sun, Tingrong Luo, Wenjun Liu, Jing Li

**Affiliations:** 1 State Key Laboratory for Conservation and Utilization of Subtropical Agro-Bioresourses & Laboratory of Animal Infectious Diseases, College of Animal Sciences and Veterinary Medicine, Guangxi University, Nanning, Guangxi, China; 2 CAS Key Laboratory of Pathogenic Microbiology and Immunology, Institute of Microbiology, Chinese Academy of Sciences, Beijing, China; 3 University of Chinese Academy of Sciences, Beijing, China; 4 Institute of Microbiology, Center for Biosafety Mega-Science, Chinese Academy of Sciences, Beijing, China; University of Tennessee Health Science Center, UNITED STATES

## Abstract

Influenza B virus (IBV) belongs to the *Orthomyxoviridae* family and generally causes sporadic epidemics but is occasionally deadly to individuals. The current research mainly focuses on clinical and pathological characteristics of IBV. However, to better prevent or treat the disease, one must determine the strategies developed by IBV to invade and disrupt cellular proteins and approach to replicate itself, to suppress antiviral innate immunity, and understand how the host responds to IBV infection. The B/Shanghai/PD114/2018 virus was able to infect alveolar epithelial cells (A549) cells, with good potential for replication. To identify host cellular responses against IBV infection, differentially expressed genes (DEGs) were obtained using RNA sequencing. The GO and KEGG pathway term enrichment analyses with the DEGs were performed, and we found that the DEGs were primary involved in metabolic processes and cellular function, which may be related to the host response, including the innate immune response against the virus. Our transcriptome analysis results demonstrated robust induction of interferon and interferon-stimulated gene expression by IBV in human cells during the early stages of infection, providing a foundation for further studies focused on antiviral drug development and interactions between the virus and host.

## Introduction

Influenza viruses are single-stranded negative-sense RNA viruses belonging to the family Orthomyxoviridae, and are phylogenetically grouped into four virus genera: influenza A, B, C, and D viruses [1, 2]. Influenza B virus (IBV) generally causes local mild epidemics, but it may be fatal to individuals, especially infants, pregnant woman, the elderly, and people with impaired immune systems [3]. Since the 1980s, IBV have two genetically and antigenically differentiated lineages, termed B/Yamagata lineage and B/Victoria lineage, and the antigenic drift rate is slower compared with influenza A viruses[4]. IBV is a pathogen with very limited host range, and its natural host is mainly humans with fever and cold symptoms. However, seasonal pandemics can break out, such as from the winter of 2017 to the spring of 2018, when the Yamagata lineage strain became the dominant pandemic strain alongside IAV (H1N1 and H3N2) [5].

When influenza virus invades a host cell, the innate immune system is activated against the virus. During the infection, viruses are recognized by pattern recognition receptors, the innate response is triggered, and interferons (IFNs) are secreted to limit early viral proliferation [6]. This identification leads to an appropriate antiviral response and to the activation of inflammatory response and adaptive immune responses [7]. While the reasons for the limited host range of IBV remain unclear, one of explanations is that the interaction between IBV and the innate immune system imbues the virus with distinct characteristics, such as the role of ISG15, a typical interferon-induced antiviral gene for the innate immune response [8, 9]. Existing studies describe the production of host cytokines, including IFNs, IFN-stimulated genes (ISGs), and proinflammatory cytokines against influenza virus infection, as integral parts of the process of the cellular antiviral response [10–13]. We have also reported that type I IFNs, ISGs, and proinflammatory cytokines can be induced by IBV virions at the early stage of virus infection [7, 14].

The interaction between influenza virus infection and the host anti-viral response has been investigated in depth [15, 16], and the regulatory mechanism of IBV infection is also under investigation. Previous research has already shown that IBV ribonucleoprotein rapidly activates TLR signaling pathways [14]. Nevertheless, the overall circumstances of the innate immune response, the pathways and types of IFNs that are dominant in innate immunity against IBV infection, and how they are regulated remain unclear.

As has been demonstrated, the potential cytokines after viral infection can be directly or indirectly identified with the aid of transcriptome analysis [17]. RNA sequencing (RNA-seq) technology has been used to identify differences in gene regulation, which links transcriptional activators to the general transcriptional machinery [18]. In the present study, we investigated the host immune responses against IBV infection, using the B/Shanghai/PD114/2018 strain as a model, to study the anti-viral capability of IFNs and ISGs, which released in response to IBV infections. These results give us a glimpse into the host innate immunity and antiviral responses, particularly, multiple signaling pathways and antiviral factors that collectively limit viral replication.

## Materials and methods

### Cell lines, antibodies, and virus preparation

Human lung carcinoma epithelial cells (A549) and Madin-Darby Canine Kidney cells (MDCK) were obtained from the American Type Culture Collection (ATCC, Manassas, VA) and were grown at 37°C in a humidified atmosphere with 5% CO_2_ in Dulbecco’s modified Eagle’s medium (DMEM; Gibco) supplemented with 10% (v/v) fetal bovine serum (Gibco), 100 U/mL penicillin, and 100 μg/mL streptomycin.

A rabbit polyclonal antibody against NP was purchased from Thermo-Fisher Scientific. Goat anti-rabbit IgG conjugated to fluorescein isothiocyanate (FITC) were purchased from Zhongshan Golden Bridge Biotechnology (Beijing, China). The mouse anti-GAPDH monoclonal antibody and anti-GFP antibody were purchased from Santa Cruz Biotechnology. HRP goat anti-mouse IgG was purchased from Beyotime.

Genes encoding full-length ISGs, including the IFIT family (*IFIT1*, *IFIT2*, *IFIT3*, and *IFIT5*), IFITM family (*IFITM1*, *IFITM2*, *IFITM3*, and *IFITM5*), *ISG15*, *ISG20*, *LAMP3*, and *RSAD2*, were synthesized by GENEWIZ and then cloned into the pcDNA3.1-GFP vector.

The B/Shanghai/PD114/2018 (B-SH; Yamagata lineage) virus was isolated from patients in Shanghai, China, and was a gift from Prof. Dayan Wang of the Chinese Center for Disease Control and Prevention. The virus stock was propagated in the allantoic cavities of 9-day-old specific-pathogen-free embryonated chicken eggs, and the virus titer was determined by 10-fold serial titration in MDCK cells according to plaque assays. The virus was stored at −80°C before use.

### Plaque assay

Plaque assays were performed as previously described [19]. Briefly, MDCK cells were plated on 6-well culture plates and infected by serially diluted B-SH virus in serum-free DMEM supplemented with 2 μg/mL of TPCK-treated trypsin for 1 h at 37°C, 5% CO_2_. Then, the virus was removed, and the cells were washed with sterile phosphate-buffered saline (PBS). Next, the cells were overlaid with phenol red free-DMEM medium containing TPCK-treated trypsin (2 μg/mL), 0.6% low melting point agarose and incubated at 37°C with 5% CO_2_. Visible plaques were counted 72 h post infection to determine the virus titer. Data of all assays were obtained from at least three independent experiments, and expressed as means ± SD.

### Immunofluorescence assay

A549 cells were seeded in 24-well culture plates overnight. The cells were proliferated on glass coverslips, infected with B-SH (MOI = 0.1) until a monolayer of cells spread to > 80% of the bottom of the culture glass coverslips, incubated for 1 h, and then the inoculum was displaced with DMEM for continued incubation at 37°C, 5% CO_2_. Cells were collected at 0, 4, 8, and 12 h, and the specific steps of immunofluorescence assay, as described by Jing et al [7] and Cao *et al* [15].

### *In vitro* infection

B-SH virus titer was quantified by a plaque formation assay. When the A549 cells density reached 90%, the cells were infected with B-SH (MOI = 0.1), and were cultured for 4 h in serum-free DMEM medium. Viral inoculum was removed, cells were washed with PBS, and fresh DMEM medium was added. After infection for 0, 4, 8, and 12 h, cells were collected for total RNA extraction.

### Total RNA extraction

Total RNA was extracted from cells using the TRIzol^®^ Reagent according to the manufacturer’s protocol, and the further detailed in reference [7]. The RNA quality was measured by using a NanoDrop ND1000 spectrophotometer (Thermo-Fisher Scientific). Only high-quality RNA samples (OD value = 1.8–2.2, and RIN value ≥ 6.5) were used for constructing viral cDNA libraries.

### Library preparation and Illumina Hi-seq xten sequencing

The libraries for RNA-Seq were prepared using a TruSeqTM RNA sample preparation Kit according to the manufacturer’s protocol (Illumina, San Diego, CA, USA). At first, messenger (poly A+) RNAs were purified using poly A tract, fragmented into short pieces with fragmentation buffer. Second, the double-stranded cDNA were synthesized using random hexamers with mRNA fragments as template, followed by end repair, and phosphorylation. Then, the cDNA fragments of 200-300bp were selected from the Agarose for PCR amplification to construct cDNA library. The cDNA library were quantified with TBS380, and the RNA-seq date were sequenced with the Illumina HiSeq xten platform by Majorbio Bio-Pharm Technology Co., Ltd with a read length of 150 nt.

### Read mapping

The raw paired-end reads was trimmed and the quality control was performed using Sickle (https://github.com/najoshi/sickle) and SeqPrep (https://github.com/jstjohn/SeqPrep) [20]. Gene regions were expanded following read depth, and the operon was obtained. In addition, the whole genome was split into multiple 15,000-bp windows that share 5,000 bp. The latest human reference genome (GRCH38) was used in fasta format for the RNAseq analysis.

### Gene ontology (GO) and Kyoto Encyclopedia of Genes and Genomes (KEGG) enrichment analysis of DEGs

Gene Ontology (GO) enrichment analysis of DEGs was performed using GOatools database (http://deweylab.biostat.wisc.edu/rsem/ [21]; http://www.bioconductor.org/packages/2.12/bioc/html/edgeR.html [22]). GO functional classifications were defined using the Blast2GO software (https://github.com/tanghaibao/Goatools), with gene products classified and annotated on the basis of Biological Process (BP), Molecular Function (MF), and Cellular Component (CC). KEGG pathway analysis was implemented using the KOBAS software(http://kobas.cbi.pku.edu.cn/home.do) [23]

### Validation of RNA-seq data by qRT-PCR and Western blotting

The total RNA was extracted from human A549 cells, reverse-transcribed into cDNA, and real-time PCR was performed to examine mRNA levels using SYBR green. GAPDH genes served as internal control. The qRT-PCR primers are shown in S1 Table. Western blot analysis was used to determine the NP protein levels. Each assay was performed in triplicate.

### Statistical analyses

The data statistical method was strictly implemented according to the biostatistics. A false discovery rate (FDR) of 0.05 and a fold-change of ≥ 2 were considered significant difference. Statistical significance between experimental groups and control groups (*p* < 0.05), and the data were analysed the graphs were created with Prism 6.

## Results

### Replication of the B/Shanghai/PD114/2018 strain in A549 cells

**Fig 1:**
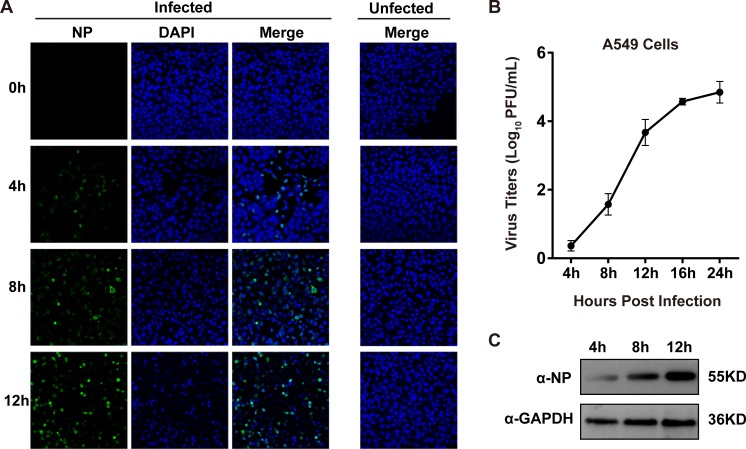
The replication of B/Shanghai/PD114/2018 (B-SH) virus in A549 cells. (A) Immunofluorescence analysis of NP expression in A549 cells. The A549 cells were infected with the B-SH virus (MOI = 0.1) virus, and NP expression was detected with a rabbit polyclonal antibody and FITC-conjugated anti-rabbit IgG antibody; the cell nucleus was countstained with DAPI. (B) Growth curve of B-SH virus. The A549 cells in 12-well plates were infected with B-SH virus (MOI = 0.1), and cell culture supernatants were collected at designated time points. The viral titers were identified on MDCK cells by plaque assays. (C) Western blotting analysis of NP expression. The A549 cells were infected with B-SH virus (MOI = 0.1) and collected 4, 8, and 12 h post infection. Then, the cells were lysed, and GAPDH was used as a control.

The B-SH viruses, which widely circulated in China from September 2017 to April 2018, especially in the autumn and winter when influenza is most prevalent, are nearly major pandemic strains. The B-SH viruses were the dominant strains isolated during the outbreak. To determine the replication efficiency of B-SH viruses in human cells, and the cells were then infected with B-SH viruses (MOI = 0.1). The expression of NP was determined by immunofluorescence, and the fluorescence intensity gradually increased with infection time. The fluorescence intensity was the most obvious at 12 h post infection, with nearly 80% of cells infected by B-SH (Fig 1A). Aliquots of cell supernatants were harvested at 4, 8, 12, 16, and 24 h post-infection (hpi). Plaque assays were used to determine viral titers, and Western blotting was used to confirm. Analysis of viral replication in A549 cells found that propagation of B-SH had reached a peak value over a 24 h period, when the titer reached 5 log_10_ PFU/mL (Fig 1B). The same results were verified by Western blotting (Fig 1C).

### Summary of trimming and read mapping data generated in A549 cells

Total RNA was extracted from infection cells, cDNA libraries were created and sequenced on Illumina HiSeq 4000, three biological replicates for three different sets of cell samples and time points were used (Table 1). We mapped the trimmed reads to the human reference genome database, and the uniquely mapped sequence reads were further analyzed. Approximately 61.6 and 62.9 million raw reads were obtained from the negative control (NC) and 12 h groups, respectively. Finally, the reads were mapped to the clean experimental reads and control reads pools.

**Table 1:** Summary of trimming and read mapping data of the sequences generated in A549 cells infected with or without B-SH viruses.

Sample	Raw reads	Clean reads	Total mapped	Multiple mapped	Uniquely mapped
NC-12 h-1	61461428	60865830	58874095 (96.73%)	2072988 (3.41%)	56801107 (93.32%)
NC-12 h-2	63969408	63368968	61147321 (96.49%)	2668482 (4.21%)	58478839 (92.28%)
NC-12 h-3	56294260	55744538	53820908 (96.55%)	1806052 (3.24%)	52014856 (93.31%)
B-SH-12 h-1	64487840	63796824	57149399 (89.58%)	1863084 (2.92%)	55286315 (86.66%)
B-SH-12 h-2	60654144	60082474	53985353 (89.85%)	1727904 (2.88%)	52257449 (86.98%)
B-SH-12 h-3	63594534	62983628	56532950 (89.76%)	1818329 (2.89%)	54714621 (86.87%)

NC-12 h represents A549 cells without B/Shanghai/PD114/2018 (B-SH; Yamagata lineage) infection; B-SH-12 h represents A549 cells with B-SH infection for 12 h at a MOI of 0.1. Three replicates of NC-12 h (NC-12 h-1, -2 and -3) and B-SH-12 h (B-SH-12 h-1, -2 and -3) were analyzed by RNA-seq analysis. Multiple mapped: Statistics of the number of sequences with multiple comparison locations on the reference sequence. Uniquely mapped: Statistics of the number of sequences with unique comparative location on the reference sequence.

### Global changes in expression in response to B-SH infection

**Fig 2:**
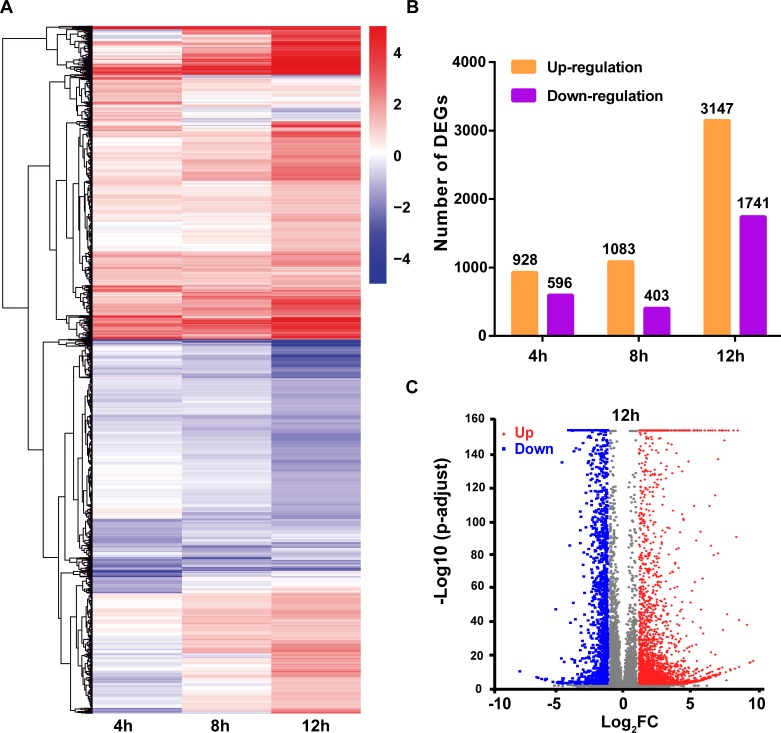
Global analysis of the differential expressed genes (DEGs) upon B-SH virus infection. (A) A549 cells were infected with B-SH virus (MOI = 0.1), and total RNA was extracted at different time points after infection for RNA-seq analysis. The heat map shows the relative gene-expression levels, with red for high levels expression and blue for low levels expression (log_2_ scale, from -4 to +4). (B) The number of DEGs at different infection time points. Shades of yellow and purple represent up-regulated and down-regulated DEGs, respectively. (C) Volcano diagram of DEGs between the NC-12 h and B-SH-12 h samples. The horizontal axis indicates expression changes of the DEGs, and the vertical axis indicates statistical test value of gene expression change differences (-log_10_ *p*-adjusted). Splashes indicate different specific genes, among which red dots show significantly up-regulated genes, and the blue dots show down-regulated genes.

As shown in Fig 2A, a heatmap was employed to analyze DEGs at the different time points. As the infection progressed, the number of DEGs gradually increased (Fig 2B), with a total of 3147 up-regulated and 1741 down-regulated genes in infected A549 cells at 12 h, and the DEGs between NC-12 h and IBV-12 h are shown in volcano plots (Fig 2C). These results indicate that the response to viral infection was strongly increased at 12 h, with some shared alternations in expression profiles at all points in time.

### Functional analysis at 12 hpi based on DEGs

**Fig 3:**
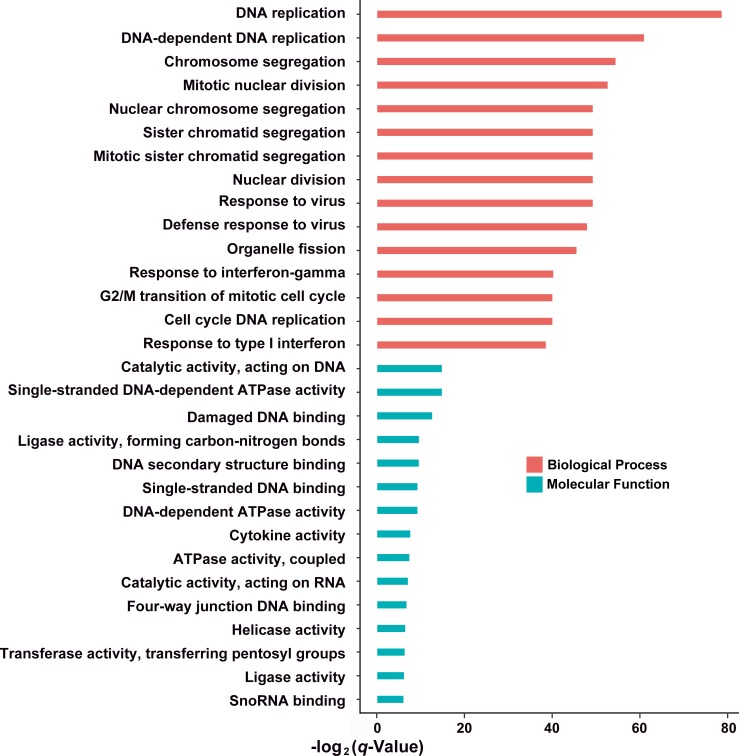
Gene ontology (GO) enrichment based on DEGs in B-SH-infected cells. The DEGs in B-SH-infected cells matched various GO terms, including the most enriched biological process (BP) and molecular function (MF), as judged by their q-values.

GO analysis revealed that the DNA replication, chromosome segregation, mitotic nuclear division were most significantly regulated by IBV-Yamagata infection, a large cluster of DEGs involved in nuclear chromosome segregation, sister chromatid segregation were observed. In addition, the catalytic activity, acting on DNA, single-stranded, DNA-dependent ATPase activity, damaged DNA binding, ligase activity, forming carbon-nitrogen bonds, and DNA secondary structure binding processes were subclassed to molecular functions (Fig 3).

**Fig 4:**
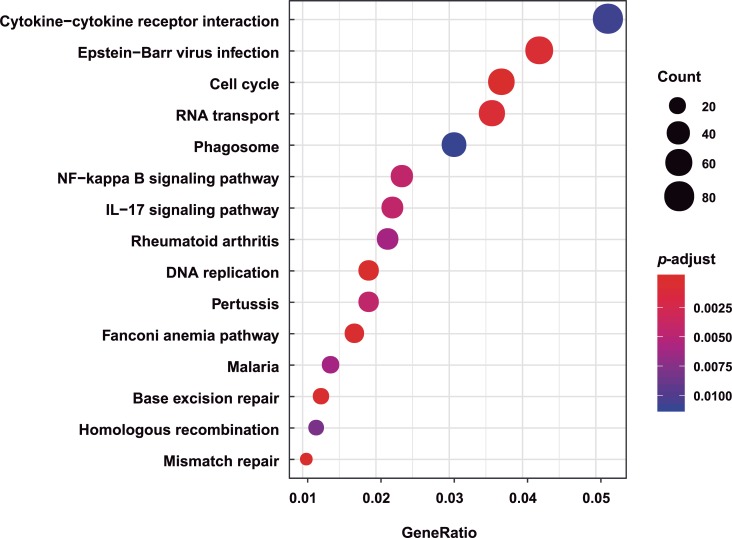
Top 15 enriched pathways of unique DEGs in A549 cells. Functions of DEGs were further annotated using Kyoto Encyclopedia of Genes and Genomes (KEGG). GeneRatio is the ratio of the number of DEGs that match a specific pathway term in the total DEGs.

Then, we specifically mapped the DEGs to KEGG pathways database terms (Fig 4). Results suggested that hundreds of KEGG pathways were readily found, among which 30 pathways in A549 cells were significantly altered (*p* < 0.01). We found that several innate immune response pathways were enriched, including NF-kB signaling pathway, cytokine-cytokine receptor, and the IL-17 signaling pathway, indicating that the natural immune response plays a significant role in host resistance to IBV infection.

As shown in Fig 4, RNA transport, DNA replication, homologous recombination, base excision repair, and mismatch repair are affiliated with genetic information processing; cell cycle is affiliated with cellular processes; and Epstein-Barr virus infection and malaria are affiliated with human disease, all of which were also enriched. Epstein-Barr virus infection was significantly enriched, suggesting that the host response related to innate immunity caused by IBV infection might be similar to that caused by Epstein-Barr virus infection. The analysis results of the top 15 hits suggest that hosts develop a rapid and robust innate immune response to fight IBV infection, which is consistent with the results of GO enrichment analysis.

### Type III IFN is extremely elevated and peaks at 12 h after infection

The GO enrichment analysis of DEGs showed that DNA replication, chromosome segregation, and mitotic nuclear division were the main types among biological process, indicating that these A549 cell processes were most influenced by IBV-Yamagata infection. In addition, response to virus and the defense response to virus, which are related to host innate immunity, were also enriched. Further analysis found that IFNs and ISGs, especially type III IFNs (L1, L3, and L2) and ISGs such as the IFIT family (*IFIT1*, *2*, *3*, and *5*), IFITM family (*IFITM1*, *2*, *3*, and *5*), and *ISG15*, *ISG20*, *LAMP3*, and *RSAD2*, were included (Table 2). IFN-L1, IFN-L3, and IFN-L2 were the top three DEGs with the highest up-regulation fold changes, at 1172-, 723-, and 637-fold (S2 Table). The type III IFNs were significantly up-regulated in A549 cells, further demonstrating that IFN-Ls are the chief regulator cytokines of anti-IBV responses.

**Table 2:** Gene ontology (GO) enrichment for differentially expressed genes (DEGs) upon IBV-Yamagata infection in A549 cells.

Top	Description	Name of differentially expressed genes
1	DNA replication	*BRCA1/MCM10/SMC1A/MCM2/MCM6/FGFR1/SUPT16H/CDC6/MCM5/MCM4/GRWD1/TIMELESS/MCM3/CACYBP/DHX9/CCNA2/SSRP1/MCM7/RRM1/FEN1/CDK1/RBM14/GINS1/CHAF1A/NUCKS1/RPA1/POLD1/CLSPN/POLE/POLA1/SAMHD1/RFWD3/NASP/DTL/POLA2/TOPBP1/RECQL4/EXO1/GINS4/RFC5/BCL6/TONSL/GINS2/POLE3/ALYREF/POLD2/MCMBP/CDC45/NUP98/GTPBP4/BLM/STOML2/RNASEH2A/DNA2/CDK2/ESCO2/PDS5A/RFC2/DSCC1/TICRR/CENPX/DNAJA3/RFC4/SMC3/TREX1/PRIM1/GMNN/CHAF1B/RFC3/GEN1/CHTF18/FBXO5/RBBP8/ORC1/DUT/MCM8/GINS3/PCLAF/RPA2/MMS22L/SLBP/E2F8/POLD3/CHEK1/POLE2/TIPIN/RAD51/DBF4/CCNE1/E2F7/DBF4B/ORC6/PRIM2/CDC7/ATAD5/STN1/RAD1/ORC3/FANCM/POLE4/RMI1/ZPR1/CDT1/ORC5/CENPS/SLX4/RPA3/THOC1/NFIX/PTMS/DONSON/CCNE2/CDAN1/BARD1/HRAS/GLI2/PRIMPOL/ACVRL1/ZRANB3/ACHE/NT5M/AICDA*
2	DNA-dependent DNA replication	*MC1A/MCM2/MCM6/FGFR1/CDC6/MCM5/MCM4/MCM3/MCM7/FEN1/GINS1/NUCKS1/RPA1/POLD1/POLE/POLA1/SAMHD1/RFWD3/POLA2/GINS4/RFC5/BCL6/TONSL/GINS2/POLE3/ALYREF/POLD2/MCMBP/BLM/STOML2/DNA2/CDK2/RFC2/DSCC1/TICRR/CENPX/DNAJA3/RFC4/SMC3/PRIM1/GMNN/RFC3/GEN1/CHTF18/FBXO5/GINS3/RPA2/MMS22L/SLBP/E2F8/POLD3/POLE2/TIPIN/RAD51/CCNE1/E2F7/DBF4B/PRIM2/CDC7/ATAD5/FANCM/POLE4/ZPR1/CDT1/CENPS/RPA3/THOC1/DONSON/CCNE2/PRIMPOL/ZRANB3/AICDA*
3	Chromosome segregation	*NCAPD2/BRCA1/TACC3/TRIP13/SMC1A/SPAG5/KIF22/BIRC5/KIF4A/CDC6/FAM83D/KPNB1/SMC4/ECT2/CDC20/CENPF/ZWINT/HJURP/TOP2A/RAN/CCNB1/KIF23/MKI67/INCENP/BUB1B/RACGAP1/PLK1/FEN1/BUB1/PRC1/NCAPG/NCAPD3/DLGAP5/NCAPH/CDCA5/SMC2/RCC1/MAD2L1/SPDL1/RAD21/NDC1/CDCA2/NUSAP1/KIF14/AURKB/NAA50/ACTR3/MCMBP/CENPN/NUP62/KIFC1/ESPL1/KIF2C/CENPE/BUB3/KNL1/FANCD2/CDCA8/TTK/NEK2/PDS5A/SKA3/RCC2/DSCC1/RRS1/CENPX/XRCC3/SGO2/SMC3/NUF2/KNSTRN/GEN1/KIF18A/FBXO5/CTCF/MLH1/CDK5RAP2/REC8/RAD18/RAD51C/SKA2/SGO1/NUP37/SLF1/SKA1/HASPIN/NDC80/SLF2/FANCM/NUP43/GEM/NCAPH2/CDT1/CEP85/PMF1/CENPS/PINX1/ZW10/CHAMP1/CENPQ/PSRC1/AGO4/CENPW/BEX4/DERPC/TTN/DMC1/TEX15/KIF4B/KIF25/MAPK15/SYCE1L/SYCE3*
4	Mitotic nuclear division	*NCAPD2/ANLN/TACC3/TRIP13/SMC1A/SPAG5/KIF22/AURKA/TPX2/KIF4A/CDC6/MYBL2/KPNB1/SMC4/CDC20/CENPF/ZWINT/RAN/CCNB1/KIF23/KIF11/MKI67/INCENP/EPS8/BUB1B/RACGAP1/PLK1/UBE2C/FLNA/PRC1/NCAPG/NCAPD3/DLGAP5/NCAPH/CDCA5/SMC2/RCC1/KNTC1/MAD2L1/SPDL1/HSPA1A/RAD21/NUSAP1/KIF14/AURKB/NAA50/NUP62/KIFC1/ESPL1/KIF2C/PRMT5/PKMYT1/CENPE/BUB3/KIF20B/CDCA8/TTK/NEK2/PDS5A/DSCC1/EDN1/RRS1/XRCC3/SMC3/KNSTRN/GEN1/KIF18A/BCCIP/FBXO5/CDK5RAP2/SGO1/CHEK1/AAAS/SLF1/CLASP2/HASPIN/NDC80/SLF2/NCAPH2/CDT1/CEP85/PINX1/ZW10/UBE2S/CHAMP1/BTC/BORA/MTBP/SPHK1/CDC25C/PSRC1/RGCC/INSR/DERPC/TTN/IL1B/IL1A/KIF4B/BMP7/KIF25/EGF*
5	Nuclear chromosome segregation	*NCAPD2/TACC3/TRIP13/SMC1A/SPAG5/KIF22/KIF4A/CDC6/FAM83D/KPNB1/SMC4/ECT2/CDC20/CENPF/ZWINT/RAN/CCNB1/KIF23/INCENP/BUB1B/RACGAP1/PLK1/FEN1/BUB1/PRC1/NCAPG/NCAPD3/DLGAP5/NCAPH/CDCA5/SMC2/MAD2L1/SPDL1/RAD21/NDC1/NUSAP1/KIF14/AURKB/NAA50/ACTR3/MCMBP/NUP62/KIFC1/ESPL1/KIF2C/CENPE/BUB3/KNL1/FANCD2/CDCA8/TTK/NEK2/PDS5A/RCC2/DSCC1/RRS1/CENPX/XRCC3/SGO2/SMC3/KNSTRN/GEN1/KIF18A/FBXO5/CTCF/MLH1/CDK5RAP2/REC8/RAD51C/SGO1/SLF1/HASPIN/NDC80/SLF2/FANCM/GEM/NCAPH2/CDT1/CENPS/PINX1/ZW10/CHAMP1/CENPQ/PSRC1/AGO4/DERPC/TTN/DMC1/TEX15/KIF4B/KIF25/MAPK15/SYCE1L/SYCE3*
6	Sister chromatid segregation	*NCAPD2/TACC3/TRIP13/SMC1A/SPAG5/KIF22/KIF4A/CDC6/KPNB1/SMC4/CDC20/CENPF/ZWINT/RAN/CCNB1/KIF23/INCENP/BUB1B/RACGAP1/PLK1/FEN1/BUB1/PRC1/NCAPG/NCAPD3/DLGAP5/NCAPH/CDCA5/SMC2/MAD2L1/SPDL1/RAD21/NUSAP1/KIF14/AURKB/NAA50/MCMBP/NUP62/KIFC1/ESPL1/KIF2C/CENPE/BUB3/CDCA8/TTK/NEK2/PDS5A/DSCC1/RRS1/XRCC3/SGO2/SMC3/KNSTRN/GEN1/KIF18A/FBXO5/CTCF/CDK5RAP2/RAD51C/SGO1/SLF1/HASPIN/NDC80/SLF2/NCAPH2/CDT1/PINX1/ZW10/CHAMP1/PSRC1/DERPC/TTN/KIF4B/KIF25/MAPK15*
7	Mitotic sister chromatid segregation	*NCAPD2/TACC3/TRIP13/SMC1A/SPAG5/KIF22/KIF4A/CDC6/KPNB1/SMC4/CDC20/CENPF/ZWINT/RAN/CCNB1/KIF23/INCENP/BUB1B/RACGAP1/PLK1/PRC1/NCAPG/NCAPD3/DLGAP5/NCAPH/CDCA5/SMC2/MAD2L1/SPDL1/RAD21/NUSAP1/KIF14/AURKB/NAA50/NUP62/KIFC1/ESPL1/KIF2C/CENPE/BUB3/CDCA8/TTK/NEK2/PDS5A/DSCC1/RRS1/XRCC3/KNSTRN/GEN1/KIF18A/FBXO5/CDK5RAP2/SGO1/SLF1/HASPIN/NDC80/SLF2/NCAPH2/CDT1/PINX1/ZW10/CHAMP1/PSRC1/DERPC/TTN/KIF4B/KIF25*
8	Nuclear division	*NCAPD2/ANLN/TACC3/ASPM/TRIP13/SMC1A/SPAG5/KIF22/AURKA/TPX2/KIF4A/CDC6/MYBL2/KPNB1/SMC4/CDC20/CENPF/ZWINT/TOP2A/RAN/CCNB1/KIF23/KIF11/MKI67/INCENP/EPS8/BUB1B/RACGAP1/PLK1/UBE2C/FLNA/PRC1/NCAPG/NCAPD3/DLGAP5/NCAPH/CDCA5/SMC2/RCC1/KNTC1/MAD2L1/CKS2/SPDL1/HSPA1A/RAD21/NDC1/NUSAP1/KIF14/AURKB/NAA50/FANCA/ACTR3/NUP62/KIFC1/ESPL1/KIF2C/PRMT5/PKMYT1/CENPE/BUB3/KIF20B/FIGNL1/FANCD2/CDCA8/TTK/NEK2/PDS5A/DSCC1/EDN1/RRS1/CENPX/XRCC3/SGO2/SMC3/KNSTRN/PSMD13/RAD54B/GEN1/KIF18A/BCCIP/FBXO5/MLH1/CDK5RAP2/REC8/RAD51C/SGO1/MYBL1/CHEK1/AAAS/SLF1/CLASP2/HASPIN/PSMC3IP/NDC80/SLF2/RAD1/FANCM/NCAPH2/CDT1/CEP85/CENPS/PINX1/ZW10/UBE2S/CHAMP1/CYP26B1/BTC/BORA/MTBP/SPHK1/CDC25C/PLCB1/PSRC1/RGCC/AGO4/INSR/DERPC/TTN/IL1B/DMC1/IL1A/TEX15/KIF4B/BMP7/KIF25/MAPK15/CNTD1/EGF/SYCE1L/SYCE3/TUBB8*
9	Response to virus	*EIF2AK2/OAS1/BNIP3L/NOP53/DDX58/OAS3/OAS2/IFIH1/STAT1/IFIT3/IFIT2/TRIM25/NT5C3A/IRF1/IFI6/ILF3/SHFL/TRIM22/OASL/DHX9/DDX60/PARP9/HERC5/PML/NLRC5/LCN2/CCT5/ADAR/IFI16/DTX3L/TRIM56/STAT2/ISG20/CFL1/IRF7/IFIT1/IFITM1/ISG15/PLSCR1/FLNA/ZC3H12A/IFI27/MX1/DDIT4/IFIT5/IFITM2/DDX21/ODC1/BANF1/STMN1/SAMHD1/SERINC5/NPC2/BIRC3/ABCE1/IRF9/CCL5/GBP3/TRIM5/CDK6/TRIM38/IFI44L/TRIM34/GBP1/IFI44/LGALS9/ZC3HAV1/PMAIP1/TNFAIP3/IFITM3/TREX1/RNF26/POLR3K/RRP1B/TLR3/POLR3G/BST2/APOBEC3F/F2RL1/IKBKE/SKP2/IL23A/IRF2/EXOSC4/UNC13D/MX2/EXOSC5/POLR3H/C1QBP/RSAD2/PDE12/BCL2L11/TMEM173/POLR3B/FOSL1/BCL2/DHX58/CD40/IFNL1/IL6/IFNL3/IFNL2/SPON2/IFNB1/CXCL10/IFNL4/RTP4/IFNLR1/IL1B/APOBEC3G/DCLK1/CCL22/IL33/CARD9/TNF/IRAK3/APOBEC3H/DMBT1/VWCE/CCL8/CCL4/AZU1*
10	Defense response to virus	*EIF2AK2/OAS1/BNIP3L/NOP53/DDX58/OAS3/OAS2/IFIH1/STAT1/TRIM25/NT5C3A/IRF1/IFI6/ILF3/SHFL/TRIM22/OASL/DHX9/DDX60/PARP9/HERC5/PML/NLRC5/ADAR/IFI16/DTX3L/TRIM56/STAT2/ISG20/IRF7/IFIT1/ISG15/PLSCR1/FLNA/ZC3H12A/IFI27/MX1/DDIT4/IFIT5/DDX21/SAMHD1/SERINC5/BIRC3/ABCE1/IRF9/GBP3/TRIM5/TRIM38/IFI44L/TRIM34/GBP1/ZC3HAV1/PMAIP1/TNFAIP3/IFITM3/TREX1/RNF26/POLR3K/TLR3/POLR3G/BST2/APOBEC3F/F2RL1/SKP2/IL23A/IRF2/EXOSC4/UNC13D/MX2/EXOSC5/POLR3H/C1QBP/RSAD2/PDE12/TMEM173/POLR3B/BCL2/DHX58/CD40/IFNL1/IL6/IFNL3/IFNL2/SPON2/IFNB1/CXCL10/IFNL4/RTP4/IFNLR1/IL1B/APOBEC3G/IL33/CARD9/APOBEC3H/DMBT1/AZU1*
11	Organelle fission	*NCAPD2/ANLN/TACC3/ASPM/TRIP13/SMC1A/SPAG5/KIF22/AURKA/TPX2/KIF4A/CDC6/MYBL2/KPNB1/SMC4/CDC20/CENPF/ZWINT/TOP2A/RAN/CCNB1/KIF23/KIF11/MKI67/INCENP/EPS8/BUB1B/RACGAP1/PLK1/STAT2/UBE2C/FLNA/PRC1/NCAPG/NCAPD3/DLGAP5/NCAPH/CDCA5/SMC2/RCC1/KNTC1/MAD2L1/CKS2/SPDL1/HSPA1A/RAD21/NDC1/NUSAP1/KIF14/AURKB/NAA50/FANCA/ACTR3/NUP62/KIFC1/ESPL1/KIF2C/PRMT5/PKMYT1/CENPE/BUB3/KIF20B/FIGNL1/FANCD2/CDCA8/TTK/NEK2/PDS5A/DSCC1/EDN1/RRS1/CENPX/XRCC3/SGO2/SMC3/KNSTRN/PSMD13/RAD54B/GEN1/KIF18A/BCCIP/FBXO5/MLH1/CDK5RAP2/REC8/RAD51C/SGO1/MYBL1/CHEK1/AAAS/SLF1/CLASP2/HASPIN/PPARGC1A/PSMC3IP/NDC80/SLF2/RAD1/FANCM/NCAPH2/CDT1/CEP85/CENPS/PINK1/PINX1/ZW10/UBE2S/CHAMP1/CYP26B1/BTC/BORA/CRYZL2P-SEC16B/MTBP/DHODH/SPHK1/CDC25C/PLCB1/MTFR2/PSRC1/RGCC/AGO4/INSR/DERPC/PRKN/TTN/IL1B/DMC1/IL1A/TEX15/KIF4B/BMP7/KIF25/MAPK15/CNTD1/EGF/SYCE1L/SYCE3/TUBB8*
12	Response to interferon-gamma	*CD44/SP100/OAS1/HSP90AB1/OAS3/OAS2/STAT1/TRIM25/IRF1/ASS1/SHFL/TRIM22/OASL/PARP9/PML/NLRC5/GBP2/B2M/PARP14/IRF7/IFITM1/HLA-C/HLA-E/HLA-A/HLA-B/HLA-F/IFITM2/CLDN1/ICAM1/IRF9/CCL5/TRIM21/ACTR3/TRIM5/TRIM38/TRIM34/NMI/GBP1/LGALS9/IFITM3/EDN1/GBP4/MT2A/TLR3/RAB43/GSN/GCH1/BST2/IRF2/TRIM31/SNCA/CXCL16/CASP1/PDE12/SLC26A6/RAB20/STXBP4/CD40/CDC42EP2/ADAMTS13/NOS2/SLC11A1/MEFV/GBP5/SOCS1/TLR4/TLR2/CCL3/UBD/PTAFR/WAS/HLA-G/TXK/IRF6/HPX*
13	G2/M transition of mitotic cell cycle	*BRCA1/HSP90AA1/AURKA/TPX2/NOP53/YWHAE/CCND1/FOXM1/CENPF/OPTN/CCNB1/APP/CALM2/CCNA2/PLK1/CDK1/CKAP5/TUBB4B/TUBB/PSMB8/MELK/CALM3/PSMD2/TUBG1/TUBA4A/PSME3/PSMD11/HMMR/DYNLL1/CLSPN/RAD21/PSMD1/CCNB2/DTL/AJUBA/KIF14/AURKB/PSME1/PSMC3/CIT/PKMYT1/PSMD14/CDK4/BLM/CDKN1A/PSMB9/CDK2/PSMC5/NEK2/RCC2/DCTN1/TICRR/PLK4/PSMD13/CEP78/BACH1/CDC25A/FBXO5/CDK5RAP2/RAD51C/NAE1/HAUS7/ODF2/DBF4B/FOXO4/CDC7/NINL/NEDD1/PSMB10/CENPJ/SSNA1/HAUS8/PINX1/PSMA8/HAUS5/WEE1/CEP135/DONSON/HAUS2/BORA/CEP41/CEP152/FGFR1OP/HAUS3/CEP72/TUBB4A/CDC25C/PLCB1/KDM8/WNT10B/STOX1/NES/ESRRB*
14	Cell cycle DNA replication	*SMC1A/FGFR1/FEN1/RPA1/POLD1/POLE/POLA1/POLA2/RFC5/BCL6/POLE3/POLD2/DNA2/RFC2/RFC4/SMC3/PRIM1/GMNN/RFC3/FBXO5/RPA2/SLBP/E2F8/POLD3/POLE2/TIPIN/RAD51/E2F7/DBF4B/PRIM2/CDC7/ATAD5/POLE4/ZPR1/CDT1/RPA3/AICDA*
15	Response to type I interferon	*SP100/IFI35/OAS1/HSP90AB1/OAS3/OAS2/STAT1/IFIT3/IFIT2/IRF1/IFI6/SHFL/OASL/NLRC5/ADAR/GBP2/TRIM56/STAT2/ISG20/MYD88/USP18/IRF7/IFIT1/IFITM1/ISG15/PSMB8/HLA-C/HLA-E/HLA-A/HLA-B/IFI27/MX1/HLA-F/IFITM2/SCRIB/SAMHD1/ABCE1/IRF9/IFITM3/TREX1/XAF1/BST2/IKBKE/IRF2/MX2/RSAD2/EGR1/IFNB1/HLA-G/IRF6*

### Validation of RNA-seq data by qRT-PCR and Western blotting

**Fig 5:**
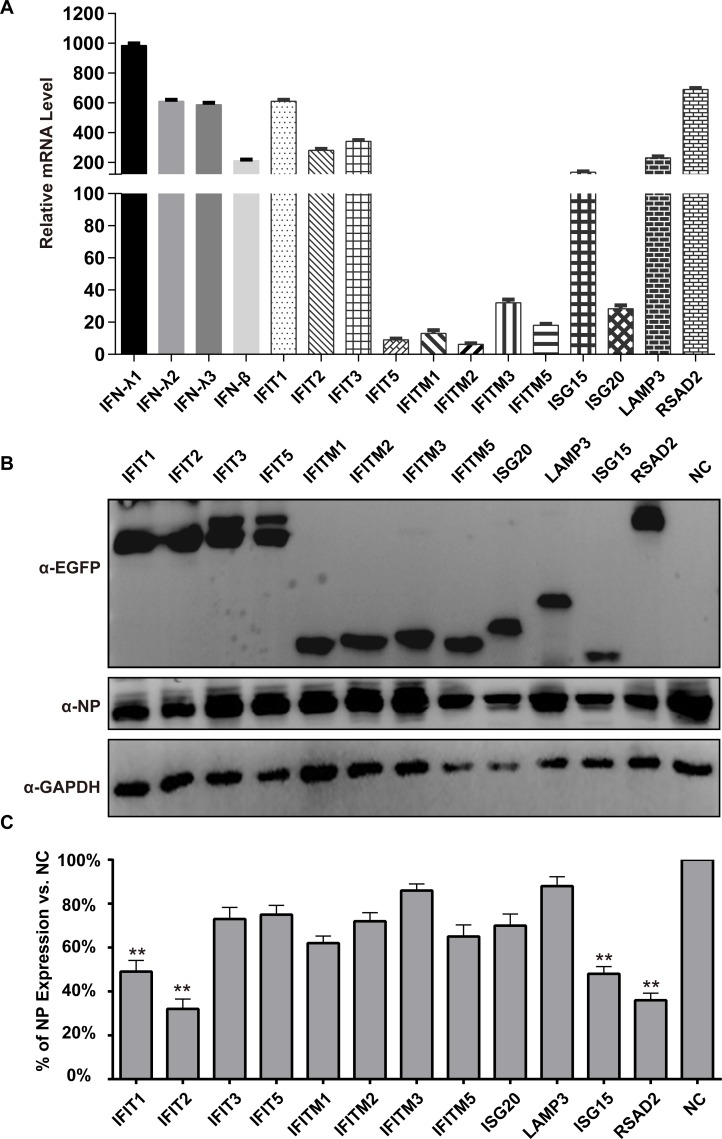
qRT-PCR and Western blotting verification of 16 DEGs. (A) Gene expression associated with DEGs was verified by RT-qPCR; RNA levels were normalized to GAPDH. Three replicates were performed, and bars represent the means ± SD (n = 3). (B) The analysis of the NP expression levels by Western blotting in different groups. The A549 cells were transfected with ISG expression plasmids for 12 h, and then the cells were infected with IBV at a MOI of 0.1 for 24 h. GAPDH was used as a control. The NP protein was identified with a rabbit anti-NP polyclonal antibody. The expression of the 12 ISGs was detected using a mouse anti-EGFP monoclonal antibody. A549 cells transfected with empty plasmid were employed as a negative control (NC). (C) The expression rates of NP were compared by gray scale analysis.

To verify the gene expression profiles from the RNA-Seq analysis, we picked several DEGs and then confirmed their expression by quantitative RT-PCR (Fig 5A) and Western blotting (Fig 5B). In addition, the intensities of the bands corresponding to the expression levels of NP protein in the different groups were scanned and analyzed, and the results indicated that *IFIT2* shows the strongest antiviral effect, followed by *RSAD2* and *IFIT1*, which inhibited the expression of NP to 68.4, 64.0, and 52.8%, respectively (Fig 5C). Thus, the expression trend of DEGs in the RNA-seq data was consistent with qRT-PCR analysis, indicating that our transcriptome sequencing data were relatively reliable.

## Discussion

Influenza B is one of the main causes of seasonal influenza, but key knowledge gaps persist, especially in the transcriptomics analyses of the pandemic strains. Our study involved a genome-wide transcriptome analysis of human cells that were infected with IBV-Yamagata. The results are in line with consensus expectations, as a large number of DEGs that are associated with IBV infection were identified, especially those related to innate immunity.

It is well known that the innate immunity is the fist line of host defense against pathogens. When a host is infected with the virus, a class of antiviral signaling molecules is immediately triggered, enhancing specific signal transduction cascades, initiating the inherent antiviral immune response, and ultimately eliminating the virus by producing necessary effector molecules, such as IFNs, interleukin, ISGs, and so on [24, 25].

Type III IFNs have antiviral effects, and the main difference between them is that IFN-L acts in mucosal tissues, including the gut, the respiratory tract, and the reproductive tract, and the antiviral capability of IFN-L is mainly limited to mucosal tissues because of the high levels of expression of the IFN-L receptor in epithelial cells [14]. Previous studies extensively demonstrate that IFN-L exerts defensive effects via mucosal barriers against enteric viruses that are susceptible to the intestinal tract, influenza A virus which targets the respiratory tract, and the Zika virus that can infect via the reproductive tract [26]. In our study, type III IFNs was also significantly up-regulated in human cells, highlighting that IFN-Ls are the chief regulatory cytokines of the antiviral response.

Type III IFNs are also related to adaptive immune responses that are initiated by mucosal infection [27]. Recent vaccination research shows that IFN-Ls can promote adaptive immune responses by boosting the release of thymic stromal lymphopoietin, stimulating adaptive immune responses [26, 28]. Animal experiments demonstrate that type III IFNs specifically enhance influenza A virus-specific IgG and IgA production when the vaccine is administered by the mucosal immune mode [29]. The same result has been achieved with HSV, where IFN-L enhances the expression of HSV-specific IgG, IFN-γ synthesis, and protection against HSV infection [26, 30]. This indicates that Type III IFNs specifically boost mucosal immunity, which should be considered to help design more efficient vaccine immunization programs against viral infections.

While a large number of host natural immune response-related pathways were enriched upon IBV infection, some pathways were involved in the regulation of viral gene replication, indicating that virus and host gene replication has important effects on each other. The NF-kB signaling pathway, cytokine-cytokine receptor, and Interleukin-17 (IL-17) signaling pathway were significantly enriched, indicating that crosstalk occurred between IFN signaling and others. However, the detailed mechanism of this crosstalk needs further research. Previous studies report that the cross-talk presumably functions as an antiviral response overall [31, 32]. IL-17 is a multifunctional cytokine whose expression and function are closely associated with host defenses against various pathogens, as well as tissue inflammation [33]. It has been reported that IL-17 is relevant to poor outcome in H1N1 influenza virus infection, but IL-17 is beneficial for eliminating virus in immune response to H5N1 influenza virus [34]. Also, IL-17 regulates the inflammatory response and decreases the symptoms of RSV-associated pulmonary inflammation [35]. However, the functions of IL-17 after infection with IBV have not been reported yet.

Previous studies demonstrate that single ISGs can inhibit virus replication, and transfecting A549 cells with a plasmid overexpressing ISG15 inhibits respiratory syncytial virus replication to inhibit viral growth [36]. Similarly, transfecting HEK293T cells with plasmids encoding RSAD2 can restrict tick-borne encephalitis virus replication [37, 38]. Overexpression system validation does provide additional explanations for the individual roles of the key genes. There are many reports in the literature, such as the overexpression of ISG15 [39, 40], and ISG15 depletion cells and ISG15 depletion mice have been used as substrates for the study of the functions of IFN-mediated antiviral effectors [41, 42]. In addition, we plan to establish stable cell lines with ISG15 and other ISG overexpression or silencing to more thoroughly investigate the functions of these genes. The replication cycle of influenza virus in A549 cells is ~8 h. The virus enters its second replication cycle 12 h after infection, and there does appear to be a mix of bystander effects.

The Epstein-Barr virus infection, rheumatoid arthritis, pertussis, and Fanconi anemia pathways were significantly enriched, indicating that there are many common characteristics between IBV and these other disease/infection models. Patients with influenza virus infections are susceptible to secondary bacterial pneumonia, including *Streptococcus* and *Klebsiella pneumoniae* [9, 43] because many bacterial adhesion molecules are up-regulated on the cell surface by influenza virus infection. The bacterial adhesion molecules are to blame for coinfection with synergistic lethality [44]. In this study, we found that the bacterial adhesion molecule genes *ITGA10*, *ITGB2*, *ITGAL*, *ITGB8*, *ITGA7*, and *ITGB4* were significantly up-regulated after IBV infection, and this partially explains why IBV is so highly mixed with bacteria. The influenza virus breaks through the first barrier of host defenses, infecting lung epithelial cells, and rapidly activating the production of pro-inflammatory cytokines [45, 46]. Our results demonstrated that a large number of inflammatory cytokine genes were up-regulated after IBV infection, such as *CXCL10*, *CD163*, and *SERPINA3*, and these inflammatory cytokines elicit significant pulmonary infiltrates and lung injury, which can further lead to complications such as fatal pneumonia.

A group of cytoskeletal proteins, including *ACTB*, *ACTL6A*, *ACTL8*, and *ARPC5L*, were down-regulated after IBV infection, indicating that actin proteins may be crucial for viral genome transcription, budding, and the release of virus particles, consistent with previous research showing that viruses invade living cells and turn them into factories to produce new progeny virions [47]. The expression of host cytoskeleton proteins is down-regulated during virus infection. The virus utilizes actin proteins for invasion, replication, and produce more virions, and evidence for this has been reported with human respiratory syncytial virus and Newcastle disease virus [48].

In our previous reports, migratory birds could also carry and spread H5N1 A/QH virus, which is likely the reasons for human infections throughout China and Europe over the past several years. We tried to analyze and compare the common genes regulated by IAV and IBV infection in A549 cells, and we found that there were very few common genes, so the infection processes of these two viruses differ [15, 49–51]. Compared to B/Victoria, there were differences between the two strains, and individual specific strains can reflect idiosyncratic specific effects. We found that the two strains have similar abilities to activate INF and ISG expression (S1–S5 Figs; S3–S5 Tables). Further, other cell types will also be investigated in our future work, to learn more about the pathogenic mechanisms of influenza virus. According to the literature, MDCK cells, human HEK 293T cells, huh7 hepatic cells [52], peripheral blood mononuclear cells [53], C1R cells [54], and HEp2 cells [55] has been widely used to the study of IBV, and these cell lines are also our reference for future studies.

Our results highlight the significance of using transcriptomics as a new approach to study of IBV-Yamagata lineage infection mechanism. The results demonstrated robust induction of IFN and ISG expression by IBV in human cells. We believe this study reveals a global and comprehensive analysis of host transcriptional changes, providing a foundation for further studies of antiviral drug development and interactions between the host and virus.

## Supporting information

S1 Fig The replication of IBV-Victoria in A549 cells. (EPS)

S2 Fig Global analysis of the differential expressed genes (DEGs) upon IBV-Victoria infection. (EPS)

S3 Fig Gene ontology (GO) enrichment based on DEGs in B/Victoria-infected cells. (EPS)

S4 Fig Top 15 enriched pathways of unique DEGs in A549 cells by B/Victoria-infected. (EPS)

S5 Fig Interferon genes were significantly up-regulated in both strains. (EPS)

S6 Fig Original underlying images for all blots. (PDF)

S1 Table List of the primer sequences used for qRT-PCR. (DOCX)

S2 Table A list of significantly up-regulated and down-regulated genes infected with influenza B/Yamagata lineage virus at 12 h post-infection. The expression of these genes was altered at least fold change by ≥ 2 folds (Significant, *p* < 0.05) compared to negative control.
(XLSX)

S3 Table A summary of our resequencing data and read mapping data were shown about A549 cells with or without IBV-Victoria infection. (DOCX)

S4 Table GO enrichment for DEGs upon IBV-Victoria infection in A549 cells. (DOCX)

S5 Table A List of significantly up-regulated and down-regulated genes infected with influenza IBV-Victoria lineage virus at 12 h post-infection. (XLS)

S1 File (DOCX)
