# Supplementary figures and images for: Robust induction of interferon and interferon-stimulated gene expression by influenza B/Yamagata lineage virus infection of A549 cells
Source: PLoS One. 2020 Apr 8;15(4):e0231039. doi: 10.1371/journal.pone.0231039 (PMC7141683; doi:10.1371/journal.pone.0231039)

**Fig 1.C**

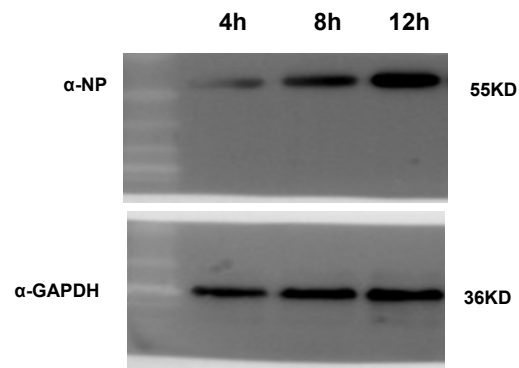

**Fig S1.C**

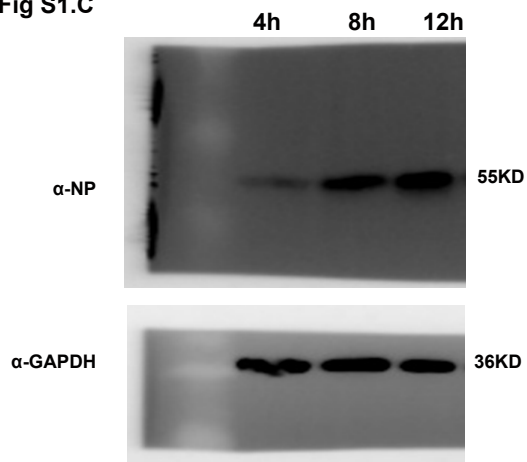

**Fig 5.A**

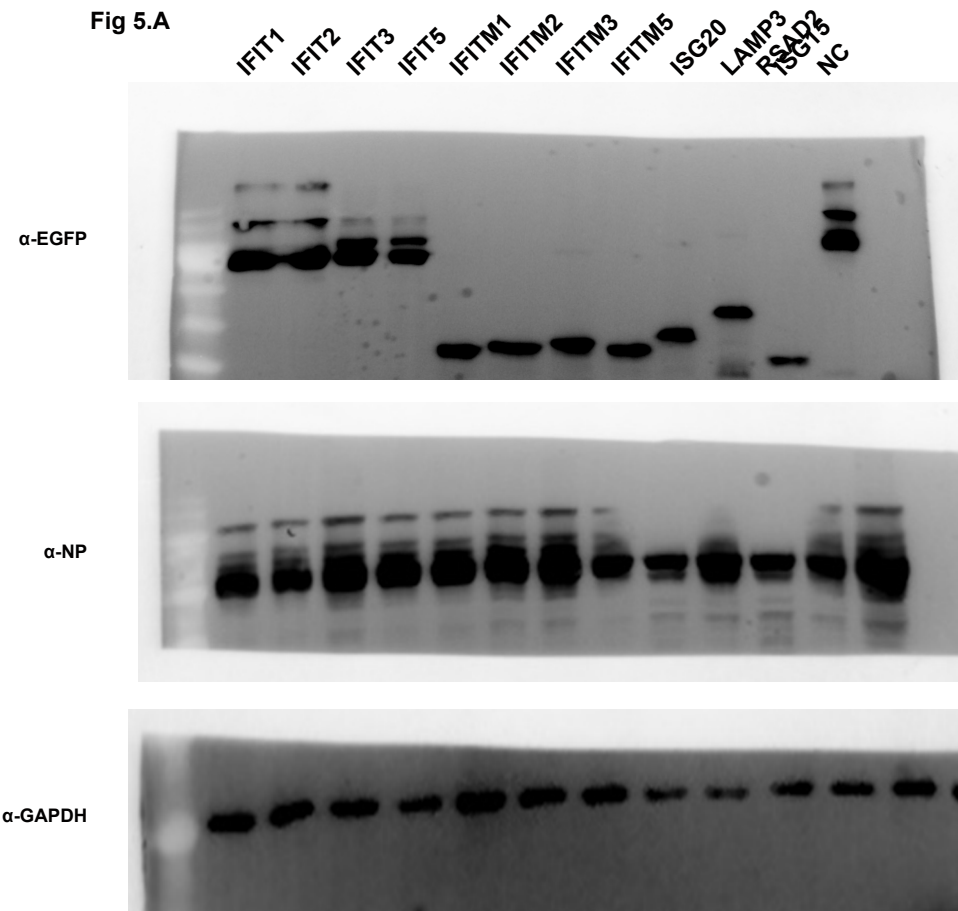

Supplement: S6 Fig — (PDF) [file pone.0231039.s006.pdf]
