# Supplementary material for: Robust induction of interferon and interferon-stimulated gene expression by influenza B/Yamagata lineage virus infection of A549 cells
Source: PLoS One. 2020 Apr 8;15(4):e0231039. doi: 10.1371/journal.pone.0231039 (PMC7141683; doi:10.1371/journal.pone.0231039)
Supplement: S1 Table — (DOCX) [file pone.0231039.s007.docx]

S1 Table. Primer sequences used for qRT-PCR.

| **Gene name** | **Forward Primer (5’-3’)** | **Reverse Primer (5’-3’)** |
| --- | --- | --- |
| IFIT1 | TTGATGACGATGAAATGCCTGA | CAGGTCACCAGACTCCTCAC |
| IFIT2 | AAGCACCTCAAAGGGCAAAAC | TCGGCCCATGTGATAGTAGAC |
| IFIT3 | TCAGAAGTCTAGTCACTTGGGG | ACACCTTCGCCCTTTCATTTC |
| IFIT5 | CGTCCTTCGTTATGCAGCCAAG | CCGTGTAGCAAAGTCCCATCTG |
| IFITM1 | AACATCCACAGCGAGACCTC | GTCCCTAGACTTCACGGAGTAG |
| IFITM2 | TTCATAGCATTCGCGTACTCC | GAATACAGGTCAAGGGCAGAG |
| IFITM3 | GAGAACCATCCCAGTAACCC | CAACCATCTTCCTGTCCCTAG |
| IFITM5 | CTGTGCTGCCTTGGTTTCCT | TTTGGAGCCATACTGCTTTG |
| IFN-λ1 | GGACGCCTTGG AAGAGTCACT | AGAAGCCTCAGGTCCCAATTC |
| IFN-λ2 | CTCAGGTTGCATGACTGGTGG | GAGGCCTCTGTCACCTTCAAC |
| IFN-λ3 | CAGCTGCAGGTGAGGGAGCGCCCCG | GGTGGCCTCCAGAACCTT |
| IFN-β | TAGCACTGGCTGGAATGAGA | TCCTTGGCCTTCAGGTAATG |
| ISG15 | CCTCTGAGCATCCTGGT | AGGCCGTACTCCCCCAG |
| 1SG20 | TGGACTGCGAGATGGTGG | GGGTTCTGTAATCGGTGAT |
| LAMP3 | AGCAAGCACCTCACCAAACTT | TGTAGTCGCTGGGGTAGTTGT |
| RSAD2 | GGTGCCTGAATCTAACCAGAAG | CCACGCCAACATCCAGAATA |
| GAPDH | GGTGGTCTCCTCTGACTTCAAGA | GTTGCTGTAGCCAAATTCGTTGT |
