# Supplementary material for: Robust induction of interferon and interferon-stimulated gene expression by influenza B/Yamagata lineage virus infection of A549 cells
Source: PLoS One. 2020 Apr 8;15(4):e0231039. doi: 10.1371/journal.pone.0231039 (PMC7141683; doi:10.1371/journal.pone.0231039)
Supplement: S3 Table — (DOCX) [file pone.0231039.s009.docx]

S3 Table. Summary of trimming and read mapping data of the sequences generated in A549 cells with or without IBV-Victoria infection.

| **Sample** | **Raw reads** | **Clean reads** | **Total mapped** | **Multiple mapped** | **Uniquely mapped** |
| --- | --- | --- | --- | --- | --- |
| NC-12h-1 | 60200210 | 59696828 | 58032375(92.12%) | 2245206(3.76%) | 55787169(93.45%) |
| NC-12h-2 | 56753540 | 56223904 | 54510112(96.95%) | 2024281(3.60%) | 52485831(93.35%) |
| NC-12h-3 | 56369180 | 55862906 | 54223971(97.07%) | 2018381(3.61%) | 52205590(93.45%) |
| B-GX-12h-1 | 49511828 | 49040542 | 31933866(65.12%) | 1525528(3.11%) | 30408338(62.01%) |
| B-GX -12h-2 | 47030488 | 46549012 | 30804219(66.18%) | 1452861(3.12%) | 29351358(63.05%) |
| B-GX -12h-3 | 53103942 | 52573708 | 35309029(67.16%) | 1681799(3.20%) | 33627230(63.96%) |

Note: NC-12 h represents A549 cells without B/Guangxi/JL1352/2018 (B-GX; Victoria lineage) infection; B-GX-12 h represents A549 cells with B-GX infection for 12 h at a MOI of 0.1. Three replicates of NC-12 h (NC-12 h-1, -2 and -3) and B-GX-12 h (B-GX-12 h-1, -2 and -3) were carried out in RNA-seq analysis.
