# Supplementary material for: Robust induction of interferon and interferon-stimulated gene expression by influenza B/Yamagata lineage virus infection of A549 cells
Source: PLoS One. 2020 Apr 8;15(4):e0231039. doi: 10.1371/journal.pone.0231039 (PMC7141683; doi:10.1371/journal.pone.0231039)
Supplement: S4 Table — (DOCX) [file pone.0231039.s010.docx]

S4 Table. Gene ontology (GO) enrichment for differentially expressed genes (DEGs) upon IBV-Victoria infection in A549 cells.

| **Top** | **Description** | **Name of differentially expressed genes** |
| --- | --- | --- |
| 1 | Protein deubiquitination | *PSMB1/RHOA/ACTB/PSME1/PSMD8/HIF1A/PSMB5/PSMA6/PSME2/NOP53/DDX58/PARK7/USP9X/TRIM21/PSMB7/PSMB6/TNIP1/UBC/UCHL1/PSMB4/ZC3H12A/PSMC3/UBB/PSMD2/EIF3F/USP18/PSMB8/UBA52/PSMB9/PSMB3/RPS27A/IFIH1/ADRM1/PSMC4/PSMC5/PSMB2/USP34/UBXN1/RUVBL1/CDC20/PSMD4/PSMA5/PSMC1/PSMA7/OTUB1/USP11/PSMF1/PSMB10/ATXN7L3/UFD1/PSMA2/PSMD13/NFKBIA/OGT/KEAP1/MDM2/NEDD8/PSMD3/PSME4/USP53/TADA3/USP47/BRCA1/EP300/TGFBR1/USP24/SIAH2/ASXL2/HCFC1/USP8/BIRC3/USP25/TNFAIP3/OTUD5/TADA2B/USP12/USP39/CLSPN/OTUD4/BABAM1/KAT2B/CDC25A/AR/JOSD2/PSMD9/EPOP/STAM2/BRCC3/USP48/USP1/TFPT/BIRC2/RHOT1/DESI2/USP15/BABAM2/BARD1/APC/CCP110/USP33/UCHL5/USP42/MDM4/ITCH/SPATA2/TAF9B/CDK1/GATA3/USP16/OTUD6B/INO80B/TRAF6/INO80C/KDM1B/USP13/USP37/ATXN7/OTULIN/SMAD4/USP28/USP54/MYSM1/MBD5/ATXN3/MAP3K7/USP21/USP46/USP49/ABRAXAS1/NFRKB/ABRAXAS2/TANK/MINDY3/INO80D/OTUD3/SENP8/SKP2/USP51/FOXO4/USP45/ADRB2/NLRP3/CCNA1* |
| 2 | Protein modification by small protein removal | *PSMB1/RHOA/ACTB/PSME1/PSMD8/HIF1A/PSMB5/PSMA6/PSME2/NOP53/DDX58/PARK7/USP9X/TRIM21/PSMB7/PSMB6/TNIP1/UBC/UCHL1/PSMB4/ZC3H12A/PSMC3/COPS6/UBB/PSMD2/EIF3F/USP18/PSMB8/UBA52/PSMB9/PSMB3/RPS27A/IFIH1/ADRM1/PSMC4/PSMC5/PSMB2/USP34/UBXN1/RUVBL1/CDC20/PSMD4/PSMA5/PSMC1/PSMA7/OTUB1/USP11/GPS1/PSMF1/PSMB10/ATXN7L3/UFD1/PSMA2/PSMD13/NFKBIA/OGT/KEAP1/MDM2/NEDD8/PSMD3/PSME4/USP53/TADA3/USP47/BRCA1/EP300/COPS3/TGFBR1/SENP6/USP24/SIAH2/ASXL2/HCFC1/USP8/BIRC3/USP25/TNFAIP3/OTUD5/TADA2B/USP12/USP39/CLSPN/OTUD4/BABAM1/KAT2B/CDC25A/AR/JOSD2/PSMD9/EPOP/STAM2/BRCC3/USP48/USP1/TFPT/BIRC2/RHOT1/DESI2/USP15/BABAM2/BARD1/APC/CCP110/USP33/UCHL5/USP42/MDM4/ITCH/SPATA2/TAF9B/CDK1/GATA3/USP16/OTUD6B/INO80B/TRAF6/INO80C/KDM1B/USP13/USP37/ATXN7/OTULIN/SMAD4/USP28/USP54/MYSM1/SENP1/MBD5/ATXN3/MAP3K7/USP21/USP46/USP49/ABRAXAS1/NFRKB/ABRAXAS2/TANK/USPL1/MINDY3/INO80D/OTUD3/SENP8/SKP2/USP51/FOXO4/USP45/ADRB2/NLRP3/CCNA1* |
| 3 | Translational initiation | *RPS20/RPL18/RPS5/EIF3I/PPP1R15A/RPLP0/EIF3L/RPL3/RPS16/RPS19/RPL18A/EIF3B/EIF3A/RPL28/RPL19/RPS13/RPS15/RPS10/RPL23/ATF4/RPL27/RPS15A/RPL35/RPS6/RPS11/RPL13A/RPL11/RPS8/RPL32/RPS3A/RPL10/RPL7A/RPS3/RPL30/RPL8/EIF4A1/RPL29/RPS14/RPL27A/RPSA/RPS9/RPS7/PPP1CA/EIF1/RPL4/RPL15/EIF3F/RPS27/EIF3K/RPS23/RPL14/RPL37A/RPL12/RPS4X/RPL10A/UBA52/RPL41/RPS18/CCL5/RPS27A/RPL35A/EIF6/EIF3G/EIF3C/RPL23A/EIF4H/RPL36/RPLP2/EIF3D/RPL5/RPS17/RPS26/EIF4EBP1/RPS21/TPR/RPL36A/RPS28/RPL21/RPL39/UHMK1/EIF2B2/RPL24/RPL9/EIF5B/RPL13/RPL38/EIF3CL/RPS29/RPS12/EIF2S1/RPLP1/RPS25/RPL31/EIF4G3/DHX29/RPS6KB1/EIF4E/EIF1AX/NCBP1/METTL3/RPS2/YTHDF3/DNAJC3/EIF2A/RPL34/RPL37/NCBP2/MTIF2/TICRR/FMR1/HSPB1/TNF/KLHL25* |
| 4 | Oxidative phosphorylation | *UQCRC1/RHOA/NDUFS8/ATP5F1B/COX6A1/PARK7/COX6B1/COX4I1/NDUFB11/ATP5F1A/STOML2/COX2/CYTB/ND2/ND5/COX1/ND3/ND4/ND1/ATP6/COX3/ND6/NDUFB9/NDUFB8/COX5B/ATP5MC3/COX8A/NDUFB10/PPIF/NDUFB2/NDUFB7/NDUFV1/UQCRH/COX5A/NDUFS7/ATP5PD/ATP5MG/ATP5F1C/NDUFA4/NDUFA13/NDUFS6/ATP5MF/CYC1/NDUFA11/NDUFA9/ATP5PO/COX7A2L/NDUFS5/NDUFA1/UQCRQ/NDUFS2/COX7C/UQCR11/UQCRFS1/NDUFAB1/MSH2/NDUFA10/ATP5MC1/NDUFS3/DNAJC30/NDUFA2/ATP5MC2/NDUFC2/BID/NDUFB4/DLD/UQCC3/NDUFA8/ATP8/NDUFC1/ATP5F1D/NDUFA3/UQCC2/SURF1/ATP7A/ND4L/NDUFB1/NDUFC2-KCTD14/CDK1/NDUFA7/UQCR10/ATPSCKMT/PDE12/NUPR1/ATP5ME/CHCHD10/SLC25A33/TEFM/DNAJC15* |
| 5 | Protein localization to endoplasmic reticulum | *RPS20/HSPA5/RPL18/RPS5/PPP1R15A/RPLP0/RPL3/CHMP4B/RPS16/RPS19/RPL18A/RPL28/RPL19/RPS13/RPS15/RPS10/RPL23/RPL27/RPS15A/OS9/RPL35/RPS6/RPS11/RPL13A/RPL11/RPS8/RPL32/RPS3A/RPL10/RPL7A/RPS3/RPL30/RPL8/RPL29/RPS14/RPL27A/RPSA/RPS9/RPS7/RPL4/RPL15/RPS27/RPS23/RPL14/RPL37A/RPL12/RPS4X/RPL10A/UBA52/RPL41/RPS18/RPS27A/RPL35A/RPL23A/RPL36/RPLP2/RPL5/RPS17/RPS26/RPS21/RPL36A/RPS28/RPL21/RPL39/SRP14/RPL24/RPL9/SRP68/RPL13/RPL38/RPS29/RPS12/RPLP1/RPS25/RPL31/GPAA1/DDRGK1/SEC62/SEC16A/SEC63/RPS2/LRRK2/RPL34/RAB3GAP2/MIA3/RPL37/TRAM2/INSIG1/SRP19* |
| 6 | Nuclear-transcribed mRNA catabolic process, nonsense-mediated decay | *RPS20/RPL18/RPS5/RPLP0/RPL3/RPS16/RPS19/RPL18A/RPL28/RPL19/RPS13/RPS15/RPS10/RPL23/RPL27/RPS15A/RPL35/RPS6/RPS11/RPL13A/RPL11/RPS8/RPL32/RPS3A/RPL10/RPL7A/RPS3/RPL30/SMG1/RPL8/RPL29/RPS14/RPL27A/RPSA/RPS9/RPS7/RPL4/RPL15/RPS27/RPS23/RPL14/RPL37A/RPL12/RPS4X/RPL10A/UBA52/RPL41/RPS18/RPS27A/RPL35A/RPL23A/RPL36/RPLP2/RPL5/RPS17/RPS26/RPS21/RPL36A/RPS28/RPL21/RPL39/RPL24/RPL9/RPL13/RPL38/RPS29/RPS12/RPLP1/RPS25/RPL31/PYM1/UPF2/NCBP1/RPS2/RPL34/SMG8/SMG9/SECISBP2/RPL37/EIF4A3/NCBP2/UPF3B/SMG6/DCP2* |
| 7 | Establishment of protein localization to endoplasmic reticulum | *RPS20/HSPA5/RPL18/RPS5/RPLP0/RPL3/CHMP4B/RPS16/RPS19/RPL18A/RPL28/RPL19/RPS13/RPS15/RPS10/RPL23/RPL27/RPS15A/RPL35/RPS6/RPS11/RPL13A/RPL11/RPS8/RPL32/RPS3A/RPL10/RPL7A/RPS3/RPL30/RPL8/RPL29/RPS14/RPL27A/RPSA/RPS9/RPS7/RPL4/RPL15/RPS27/RPS23/RPL14/RPL37A/RPL12/RPS4X/RPL10A/UBA52/RPL41/RPS18/RPS27A/RPL35A/RPL23A/RPL36/RPLP2/RPL5/RPS17/RPS26/RPS21/RPL36A/RPS28/RPL21/RPL39/SRP14/RPL24/RPL9/SRP68/RPL13/RPL38/RPS29/RPS12/RPLP1/RPS25/RPL31/SEC62/SEC63/RPS2/RPL34/RAB3GAP2/RPL37/TRAM2/SRP19* |
| 8 | SRP-dependent cotranslational protein targeting to membrane | *RPS20/RPL18/RPS5/RPLP0/RPL3/RPS16/RPS19/RPL18A/RPL28/RPL19/RPS13/RPS15/RPS10/RPL23/RPL27/RPS15A/RPL35/RPS6/RPS11/RPL13A/RPL11/RPS8/RPL32/RPS3A/RPL10/RPL7A/RPS3/RPL30/RPL8/RPL29/RPS14/RPL27A/RPSA/RPS9/RPS7/RPL4/RPL15/RPS27/RPS23/RPL14/RPL37A/RPL12/RPS4X/RPL10A/UBA52/RPL41/RPS18/RPS27A/RPL35A/RPL23A/RPL36/RPLP2/RPL5/RPS17/RPS26/RPS21/RPL36A/RPS28/RPL21/RPL39/SRP14/RPL24/RPL9/SRP68/RPL13/RPL38/RPS29/RPS12/RPLP1/RPS25/RPL31/SEC63/RPS2/RPL34/RPL37/TRAM2/SRP19* |
| 9 | Protein targeting to ER | *RPS20/HSPA5/RPL18/RPS5/RPLP0/RPL3/CHMP4B/RPS16/RPS19/RPL18A/RPL28/RPL19/RPS13/RPS15/RPS10/RPL23/RPL27/RPS15A/RPL35/RPS6/RPS11/RPL13A/RPL11/RPS8/RPL32/RPS3A/RPL10/RPL7A/RPS3/RPL30/RPL8/RPL29/RPS14/RPL27A/RPSA/RPS9/RPS7/RPL4/RPL15/RPS27/RPS23/RPL14/RPL37A/RPL12/RPS4X/RPL10A/UBA52/RPL41/RPS18/RPS27A/RPL35A/RPL23A/RPL36/RPLP2/RPL5/RPS17/RPS26/RPS21/RPL36A/RPS28/RPL21/RPL39/SRP14/RPL24/RPL9/SRP68/RPL13/RPL38/RPS29/RPS12/RPLP1/RPS25/RPL31/SEC62/SEC63/RPS2/RPL34/RPL37/TRAM2/SRP19* |
| 10 | Cotranslational protein targeting to membrane | *RPS20/RPL18/RPS5/RPLP0/RPL3/RPS16/RPS19/RPL18A/RPL28/RPL19/RPS13/RPS15/RPS10/RPL23/RPL27/RPS15A/RPL35/RPS6/RPS11/RPL13A/RPL11/RPS8/RPL32/RPS3A/RPL10/RPL7A/RPS3/RPL30/RPL8/RPL29/SSR2/RPS14/RPL27A/RPSA/RPS9/RPS7/RPL4/RPL15/RPS27/RPS23/RPL14/RPL37A/RPL12/RPS4X/RPL10A/UBA52/RPL41/RPS18/RPS27A/RPL35A/RPL23A/RPL36/RPLP2/RPL5/RPS17/RPS26/RPS21/RPL36A/RPS28/RPL21/RPL39/SRP14/RPL24/RPL9/SRP68/RPL13/RPL38/RPS29/RPS12/RPLP1/RPS25/RPL31/SEC62/SEC63/RPS2/RPL34/RPL37/TRAM2/SRP19* |
| 11 | Nuclear-transcribed mRNA catabolic process | *RPS20/RPL18/RPS5/RPLP0/RPL3/RPS16/RPS19/RPL18A/RPL28/RPL19/RPS13/RPS15/RPS10/RPL23/RPL27/RPS15A/RPL35/RPS6/RPS11/RPL13A/RPL11/RPS8/RPL32/RPS3A/RPL10/RPL7A/RPS3/RPL30/SMG1/BTG2/RPL8/RPL29/ZC3H12A/RPS14/RPL27A/RPSA/RPS9/RPS7/RPL4/RPL15/RPS27/RPS23/RPL14/RPL37A/RPL12/RPS4X/RPL10A/UBA52/RPL41/RPS18/RPS27A/RPL35A/RPL23A/RPL36/RPLP2/RPL5/RPS17/RPS26/RPS21/ZFP36/RPL36A/RPS28/RPL21/EXOSC4/RPL39/CNOT1/RPL24/RPL9/RPL13/RPL38/PELO/LSM4/RPS29/RPS12/TTC37/RPLP1/DHX36/RPS25/PATL1/DDX6/RPL31/WDR61/PYM1/DIS3/CNOT6/ATM/UPF2/NCBP1/TUT4/RPS2/EXOSC5/RPL34/EXOSC1/SMG8/SMG9/NT5C3B/SECISBP2/RPL37/EIF4A3/NCBP2/TENT4A/LSM7/UPF3B/RC3H1/ZFP36L2/NBDY/PAN3/TENT2/TUT7/PAN2/XRN1/SMG6/NOCT/TNRC6B/TENT4B/PDE12/HBS1L/DCP2/EXOSC3/CNOT6L/DIS3L2/CNOT4/CPEB3/NANOS1* |
| 12 | RNA catabolic process | *PSMB1/RPS20/RPL18/YBX1/XPO1/RPS5/RPLP0/PSME1/HNRNPC/PSMD8/RPL3/PSMB5/PSMA6/PSME2/RPS16/RPS19/RPL18A/ZC3HAV1/RPL28/RPL19/HSPA8/RPS13/OAS2/RPS15/RPS10/RPL23/RPL27/RPS15A/PSMB7/RPL35/RPS6/LRPPRC/PSMB6/RPS11/RPL13A/RPL11/RPS8/RPL32/RPS3A/RPL10/RPL7A/RPS3/UBC/RPL30/SMG1/PSMB4/BTG2/DEDD2/RPL8/RPL29/ZC3H12A/RPS14/PSMC3/RPL27A/RPSA/UBB/RPS9/RPS7/ISG20/MYD88/RPL4/RPL15/PSMD2/HNRNPA0/RPS27/RPS23/RPL14/RPL37A/RPL12/RPS4X/RPL10A/PSMB8/HSPA1B/HSPA1A/UBA52/RPL41/RPS18/PSMB9/PSMB3/RPS27A/RPL35A/VIM/PSMC4/RPL23A/PSMC5/RPL36/RPLP2/TNPO1/RPL5/PSMB2/RNH1/YBX3/APEX1/RPS17/HNRNPU/RPS26/RPS21/ZFP36/PSMD4/PSMA5/PSMC1/SND1/PSMA7/RPL36A/TBRG4/RPS28/RPL21/EXOSC4/ALKBH5/ROCK2/PSMF1/RPL39/PSMB10/HNRNPD/PSMA2/PSMD13/SYNCRIP/CNOT1/RPL24/RPL9/HNRNPR/RNASEH2A/RPL13/MAPKAPK2/RPL38/PELO/LSM4/PSMD3/PSME4/TRIR/RPS29/RPS12/PUM2/TTC37/RPLP1/DHX36/RPS25/PATL1/DDX6/PRKCA/RPL31/CARHSP1/ROCK1/PUM1/WDR61/PYM1/DIS3/CNOT6/ATM/ZPR1/PNPT1/GTPBP1/UPF2/NCBP1/METTL3/TUT4/RPS2/IGF2BP3/YTHDF3/EXOSC5/RPL34/EXOSC1/MOV10/PSMD9/RBM24/SMG8/SMG9/NT5C3B/TARDBP/AXIN2/SECISBP2/RPL37/DNA2/EIF4A3/GIGYF2/NCBP2/TENT4A/LSM7/UPF3B/RC3H1/ZFP36L2/NBDY/PAN3/FMR1/METTL14/RNASET2/TENT2/TUT7/FASTKD1/HSPB1/PAN2/RNASEH2C/XRN1/SMG6/NOCT/ANGEL2/TNRC6B/TENT4B/PDE12/HBS1L/DCP2/EXOSC3/CNOT6L/DIS3L2/CNOT4/SLFN13/AGO4/AGO3/TNFSF13/RNASEL/TNFRSF1B/SLC11A1/CPEB3/NANOS1* |
| 13 | mRNA catabolic process | *PSMB1/RPS20/RPL18/YBX1/XPO1/RPS5/RPLP0/PSME1/HNRNPC/PSMD8/RPL3/PSMB5/PSMA6/PSME2/RPS16/RPS19/RPL18A/ZC3HAV1/RPL28/RPL19/HSPA8/RPS13/RPS15/RPS10/RPL23/RPL27/RPS15A/PSMB7/RPL35/RPS6/PSMB6/RPS11/RPL13A/RPL11/RPS8/RPL32/RPS3A/RPL10/RPL7A/RPS3/UBC/RPL30/SMG1/PSMB4/BTG2/RPL8/RPL29/ZC3H12A/RPS14/PSMC3/RPL27A/RPSA/UBB/RPS9/RPS7/MYD88/RPL4/RPL15/PSMD2/HNRNPA0/RPS27/RPS23/RPL14/RPL37A/RPL12/RPS4X/RPL10A/PSMB8/HSPA1B/HSPA1A/UBA52/RPL41/RPS18/PSMB9/PSMB3/RPS27A/RPL35A/VIM/PSMC4/RPL23A/PSMC5/RPL36/RPLP2/TNPO1/RPL5/PSMB2/RNH1/YBX3/APEX1/RPS17/HNRNPU/RPS26/RPS21/ZFP36/PSMD4/PSMA5/PSMC1/PSMA7/RPL36A/TBRG4/RPS28/RPL21/EXOSC4/ALKBH5/ROCK2/PSMF1/RPL39/PSMB10/HNRNPD/PSMA2/PSMD13/SYNCRIP/CNOT1/RPL24/RPL9/HNRNPR/RPL13/MAPKAPK2/RPL38/PELO/LSM4/PSMD3/PSME4/RPS29/RPS12/PUM2/TTC37/RPLP1/DHX36/RPS25/PATL1/DDX6/PRKCA/RPL31/CARHSP1/ROCK1/PUM1/WDR61/PYM1/DIS3/CNOT6/ATM/PNPT1/GTPBP1/UPF2/NCBP1/METTL3/TUT4/RPS2/IGF2BP3/YTHDF3/EXOSC5/RPL34/EXOSC1/MOV10/PSMD9/RBM24/SMG8/SMG9/NT5C3B/TARDBP/AXIN2/SECISBP2/RPL37/EIF4A3/GIGYF2/NCBP2/TENT4A/LSM7/UPF3B/RC3H1/ZFP36L2/NBDY/PAN3/FMR1/METTL14/TENT2/TUT7/FASTKD1/HSPB1/PAN2/XRN1/SMG6/NOCT/ANGEL2/TNRC6B/TENT4B/PDE12/HBS1L/DCP2/EXOSC3/CNOT6L/DIS3L2/CNOT4/AGO4/AGO3/TNFSF13/RNASEL/SLC11A1/CPEB3/NANOS1* |
| 14 | Viral gene expression | *RPS20/RPL18/RPS5/RPLP0/EIF3L/RPL3/RPS16/RPS19/RPL18A/EIF3B/EIF3A/RPL28/RPL19/RPS13/RPS15/RPS10/RPL23/RPL27/TRIM21/RPS15A/RPL35/RPS6/RPS11/RPL13A/RPL11/RPS8/RPL32/RPS3A/RPL10/RPL7A/RPS3/RPL30/RPL8/RPL29/RPS14/PSMC3/RPL27A/RPSA/RPS9/RPS7/RPL4/RPL15/EIF3F/RPS27/RPS23/RPL14/RPL37A/RPL12/RPS4X/RPL10A/UBA52/RPL41/RPS18/CCL5/RPS27A/RPL35A/EIF3G/RPL23A/NUCKS1/RPL36/RPLP2/EIF3D/RPL5/SEC13/RPS17/SUPT4H1/RPS26/RPS21/ZFP36/TPR/RPL36A/RPS28/IFITM3/RPL21/TRIM14/RPL39/POLR2G/GTF2F1/JUN/RANBP2/NELFE/SUPT5H/RPL24/RPL9/RPL13/RPL38/RPS29/TRIM11/RPS12/POLR2C/NUP153/RPLP1/EP300/RPS25/RPL31/POLR2A/POLR2L/USF2/POLR2J/NELFCD/TRIM27/POLR2B/NUP98/CHD1/NUP133/SP1/RPS2/NUP58/REST/RPL34/TRIM8/NUP107/POLR2I/TARDBP/POLR2H/RPL37/POLR2F/POLR2D/SEH1L/NUP160/CCNT1/CCNT2/RSF1/NUP50/TRIM32/NUP35/TRIM31/CCL3/TAF11/GTF2F2/HMGA2/NUP42/CCL4/SMARCA4* |
| 15 | Viral transcription | *RPS20/RPL18/RPS5/RPLP0/RPL3/RPS16/RPS19/RPL18A/RPL28/RPL19/RPS13/RPS15/RPS10/RPL23/RPL27/TRIM21/RPS15A/RPL35/RPS6/RPS11/RPL13A/RPL11/RPS8/RPL32/RPS3A/RPL10/RPL7A/RPS3/RPL30/RPL8/RPL29/RPS14/PSMC3/RPL27A/RPSA/RPS9/RPS7/RPL4/RPL15/RPS27/RPS23/RPL14/RPL37A/RPL12/RPS4X/RPL10A/UBA52/RPL41/RPS18/CCL5/RPS27A/RPL35A/RPL23A/NUCKS1/RPL36/RPLP2/RPL5/SEC13/RPS17/SUPT4H1/RPS26/RPS21/ZFP36/TPR/RPL36A/RPS28/IFITM3/RPL21/TRIM14/RPL39/POLR2G/GTF2F1/JUN/RANBP2/NELFE/SUPT5H/RPL24/RPL9/RPL13/RPL38/RPS29/TRIM11/RPS12/POLR2C/NUP153/RPLP1/EP300/RPS25/RPL31/POLR2A/POLR2L/USF2/POLR2J/NELFCD/TRIM27/POLR2B/NUP98/CHD1/NUP133/SP1/RPS2/NUP58/REST/RPL34/TRIM8/NUP107/POLR2I/TARDBP/POLR2H/RPL37/POLR2F/POLR2D/SEH1L/NUP160/CCNT1/CCNT2/RSF1/NUP50/TRIM32/NUP35/TRIM31/CCL3/TAF11/GTF2F2/HMGA2/NUP42/CCL4/SMARCA4* |
