# Supplementary material for: Robust induction of interferon and interferon-stimulated gene expression by influenza B/Yamagata lineage virus infection of A549 cells
Source: PLoS One. 2020 Apr 8;15(4):e0231039. doi: 10.1371/journal.pone.0231039 (PMC7141683; doi:10.1371/journal.pone.0231039)
Supplement: S1 File — (DOCX) [file pone.0231039.s012.docx]

**IMMUNOFLUORESCENCE**

**(Standard Operation Procedure 1)**

**1. Materials and Reagents**

DMEM、PBS、PBS-BT、BSA、primary antibody、secondary antibody、DAPI、nitric acid、HCl、ddH_2_O、coverglasse、24-well plate、paraformaldehyde

**2. Procedure**

**2.1 Preparing coverglasses**

2.2.1 Make up 100 ml acid solution in a large glass beaker in the hood.

*Note: The acid solution is made of 2 parts of nitric acid and 1 part of HCl, and the color is orange-red.*

2.2.2 Put the 12 mm coverglasses into the acid solution one by one so that the coverglasses are evenly washed in the acid.

2.2.3 Let the coverglasses sit in the acid for 2 h or overnight swirling occasionally.

2.2.4 Wash coverglasses with ddH_2_O until pH goes up to 7.0.

2.2.5 Wash coverglasses 3 times with ddH_2_O.

**2.2 Growing cells on coverglasses**

2.2.1 Split cells onto tissue culture dishes containing coverglasses or chambered slides.

*Notes: We usually put one coverglass in one well of the 24-well plate; or no more than 2 coverglasses in one well of the 6-well plate, or no more than 5 coverglasses in a 60 mm dish. Pipette up and down or shake the dish to make sure cells are not concentrating in the center of the dish well. Make sure there are no air bubbles between the coverglasses and the tissue culture dish.*

2.2.2 Grow cells to 70-100% confluency.

**2.3 Fixation and permeablization of the cells**

2.3.1 Transfer the coverglasses or chambered slides into another tissue plate containing sufficient methanol (-20°C stock), fix for 10 min or a couple of weeks. Alternately, transfer cells to another plate including PBS. Discard PBS, adding 200 µl PBS plus 20 µl 16% paraformaldehyde. Shaking the plate for 15 min at room temperature (RT).

2.3.2 Carefully transfer the coverglasses or chambered slides from the plates and place cell side up onto secured Parafilm. Wash the coverglasses or chambered slides with 100 µl PBS immediately after transfer, never dry the cells.

2.3.3 Add 100 µl PBS-BT solutions to the coverglasses or chambered slides, let sit for 30 min at RT to permeablize and block cells.

*Note: For stringent block, 4-6% Bovine serum albumin (BSA) can be used.*

**2.4 Staining and mounting cells**

2.4.1 Incubate cells in 40 µl primary antibody (1 µg/ml final primary antibody concentration, dilute in PBS-BT) for 30 min at RT.

2.4.2 Rinse with PBS-BT twice, and then wash with 100 µl PBS-BT twice, 5 min each.

2.4.3 Cells were incubated in 40 µl secondary antibody (1 µg/ml final secondary antibody concentration, dilute in PBS-BT) for 30 min at RT.

2.4.4 Rinse with PBS-BT twice, wash with PBS-BT once, 5 min, and then wash with PBS, 5 min.

2.4.5 Incubate cells in 40 µl DAPI (1 µg/ml final concentration, 1:1,000 dilute in PBS) for 2 min, and then wash with PBS once.

2.4.6 Add 5-10 µl mounting solution to a clean microscope slide for each coverglass, place stained coverglass cell side down onto mounting solution from one edge; allow mounting solution to cover the entire surface of the coverglass, avoiding air bubbles.

2.4.7 Let the mounting solution dry and self-seal for 30 min at RT.

**Reagents**

**PBS-T solution**

10 ml 10x PBS

3 g BSA (to 3%)

1 ml 10% Triton X-100 (to 0.1%)

ddH_2_O to 100 ml

Stored at 4 °C

**Bovine serum albumin**

Make 4-6% solution in PBS-T

**PLAQUE ASSAY**

**(Standard Operation Procedure 2)**

**1. Materials and Reagents**

DMEM、PBS、MDCK、purple crystal、12-well plate、low-melting agarose、TPCK-Trypsin

**2. Procedure**

2.1 Inoculate a 12-well plate with 1×10^5^ cells, and culture in a CO_2_ incubator overnight to grow the cells into a monolayer (over 80% coverage).

2.2 Wash the MDCK cells in the 6-well plate 3 times with PBS, and aspirate the liquid in the wells.

2.3 Add an appropriate amount of serum-free and antibiotic-free DMEM culture medium to a 1.5 mL centrifuge tube. The virus was diluted in 10-fold gradients in centrifuge tubes.

2.4 Add different dilutions of virus solution to a 12-well plate, with 3 parallel wells of each dilution, 1ml per well, leaving one well for normal cell control. Place in a 37°C incubator for 1 h.

2.5 Aspirate the virus solution and wash the 6-well plate 3 times with PBS. Remove as much liquid as possible.

2.6 Heat and melt 3% low-melting agarose in a water bath. When it is cooled to about 50°C, it is mixed with phenol red-free DMEM culture medium preheated at 37°C at a ratio of 1:1 (DMEM contains 4ug/ml TPCK-Trypsin, ie, the final concentration is 2ug/ml). Add to a 12-well plate, 1 mL per well.

2.7 Place the 12-well plate at 4°C for 10-15 minutes. After the agarose has solidified, turn the 12-well plate upside down and incubate at 37°C. Observe the cytopathic condition under the microscope.

2.8 After 2-4 days of incubation, remove the 12-well plate from the incubator, add crystal violet staining, and rinse the excess dye after 10 minutes and count.

**QUANTITATIVE REAL-TIME PCR (qRT-PCR)**

**(Standard Operation Procedure 3)**

**1. Materials and Reagents**

Cells of interest, infected with IBV, Phenol-chloroform-isoamyl alcohol (Sigma), Chloroform (Sigma), pure water, Absolute Ethanol, Isopropanol, SYBR Green qPCR Supermix UDG, qPCR primers.

**2. Equipment**

Centrifuge, DNA-free hood, Speed-vac, Heat-seal machine, qPCR machine, 96-well qPCR plates (Eppendorf), Plastic films for qPCR plates (Eppendorf), Filter tips

**3. Procedure**

**3.1 Total RNA extraction**

3.1.1. Thaw one aliquot and add 500 μl of fresh TRIzol^®^ for a 1 ml total volume.

3.1.2. Add 200 μl of chloroform and shake vigorously for 15 sec.

3.1.3. Leave for 3 min at room temperature to allow for phase separation. Centrifuge for 15 min at 12,800 × g in a microcentrifuge at 4°C.

3.1.4. Carefully collect 3/4 of the upper aqueous phase without disturbing or touching the interface or organic layer. Combine the aqueous phase with an equal volume of isopropanol. Gently invert to mix.

3.1.5. Leave for 10-20 min at -20°C for precipitation.

3.1.6. Centrifuge for 15 min at 12,800 × g in a microcentrifuge at 4°C.

3.1.7. Discard the supernatant with care not to lose the pellet.

3.1.8. Wash the pellet with 1 ml of 75% ethanol. Vortex briefly or pipette up and down the sample multiple times.

3.1.9. Centrifuge for 10 min at 12,800 × g in a microcentrifuge at 4°C.

3.1.10. Remove the supernatant and invert the tubes. Allow the pellet to air-dry until clear.

3.1.11. Resuspend the pellet in 30-40 μl of ultra-pure nuclease-free ddH_2_O.

3.1.12. Incubate at 65°C for 5 min.

3.1.13. Measure RNA concentration in a Nanodrop 1000 or equivalent.

**3.2 Reverse transcription (RT)**

3.2.1. Use 2 μg of RNA for reverse transcription.

3.2.2. Prepare the RT reaction mix in PCR tubes on ice using the High Capacity RNA-cDNA kit as follows (per sample): 2 × RT buffer 5 μl, 20 × RT Enzyme mix 0.5 μl, RNA 2 μg, nuclease-free H_2_O quantity sufficient to 10 μl.

*Note: The oligo-dT primers are included in the RT buffer.*

3.2.3. Gently mix and briefly centrifuge to collect all the components at the bottom of the tube.

3.2.4. Place into a BioRad C1000 Thermal Cycler and run program to synthesize cDNA as follow: 37°C for 60 min, 95°C for 5 min, 4°C hold.

3.2.5. Store total cDNA (T-cDNA) at -20°C until ready for qPCR analysis.

**3.3 Quantitative PCR (qPCR)**

3.3.1. qPCR reactions are performed in triplicate using specific primers and the Power SYBR^®^ Green PCR Master Mixture in a Viia7 Applied Biosystem Lightcycler or equivalent.

3.3.2. Dilute cDNA 1:40 using ddH_2_O.

3.3.3. Load the plate with 4 μl of the diluted cDNA/well in triplicate.

3.3.4. Prepare Master Mix: SYBR green (5 μl) with 1 μl of qPCR primer mix. Load the plate with Master Mix 6 μl/well.

3.3.5. Run the qPCR using the following program: Denaturation (95°C, 15 sec) ; denaturation (95°C, 5 sec), annealing (60°C 31 sec), 40 cycles.

3.3.6. Data analysis using the Delta-Delta Ct.

**WESTERN BLOTTING**

**(Standard Operation Procedure 4)**

**1. Materials**

**1.1 Apparatus**

Apparatus of SDS-PAGE, Electroblotting Apparatus, PVDF membrane, Semidry transfer apparatus, Forceps, sponge pad, scissor, gloves, small plastic or glass container, Shallow tray.

**1.2 Reagents**

30% Acrylamide: 29.2% acrylamide/0.8% bis-acrylamide in H2O, H2O-saturated isobutyl alcohol, 1 M Tris-HCl, pH 8.8; 1 M Tris-HCl, Ph6.8; 10% SDS, 25% ammonium persulfate, TEMED.

Transfer buffer: Trizma base (0.25 M), 144 g Glycine (1.92 M), pH8.3.

TBST buffer: 0.9% NaCl, 20 mM Tris-HCl, pH 7.5, and 0.05% Tween-20.

First antibody: antibody against the target protein.

Second antibody: goat anti-rabbit-HRP.

Chemiluminescence reagent.

**2. Methods**

**2.1 SDS-PAGE**

2.1.1 Prepare samples as common method, mix the extracts with 1 vol of 5 × sample buffer and boil at 95°C for 10 min.

2.1.2 Centrifuge samples at 12,000g for 5 min after boiling to pellet undissolved particles and bring down moisture from the wall of the tube into the solution.

2.1.3 Allow the samples to cool down to room temperature before loading onto a gel.

2.1.4 Set the power supply at 120 V of constant voltage and start running.

2.1.5 Stop the power supply when bromophenol blue dye reaches the bottom of the gel or a desired resolution is achieved.

**2.2 Electroblotting**

2.2.1 Place the gel along with the glass plate in a tray containing 1× transfer buffer.

2.2.2 Remove the gel gently from the glass plate. The gel usually comes off the glass plate when the glass plate is gently shaken in the transfer buffer.

2.2.3 Prepare a nitrocellulose membrane and two layers of blot paper. Each blot paper layer should be 2- to 3-mm thick. Cut the membrane and the blot paper so that their length and width are each 1 cm larger than the gel. If two gels are transferred together on the same membrane, double the area of the membrane and the blot paper. Wet the membrane and blot papers with 1× transfer buffer.

2.2.4 Sandwich the gel and the membrane between the two blot paper layers and arrange this sandwich on the anode plate of the semidry blotting apparatus.

2.2.5 After electrophoresis, remove the stacking gel along with the top 1 to 2 mm of the separating gel, and the proteins in one gel are transferred to a PVDF membrane by electroblotting using the Bio-Rad wet transfer Blotter (15 V for 50 min).

2.2.6 Stop the power supply and take off the upper electrode carefully. Remove the top blot paper and the gel. Check remaining prestained markers on the gel and transferred markers on the membrane. If most of the markers were transferred, it indicates that most of your proteins of interest were also transferred.

2.2.7 Wash the membrane briefly with TBS-T.

2.2.8 Remove TBS-T and add Ponceau S solution just enough to cover the membrane.

2.2.9 Shake the tray by hand for a couple of minutes. The protein bands should be readily visible. Visually assess the efficiency of the transfer.

2.2.10 Remove the Ponceau S solution and wash the membrane with TBS-T until the staining is completely washed off.

2.2.11 Add blocking solution and incubate at room temperature for 60 min.

**2.3 Immunodetection**

2.3.1 Remove the blocking solution and add the primary antibody diluted in blocking solution.

2.3.2 Incubate the primary antibody at room temp for 1 h or at 4°C overnight with gentle shaking.

2.3.3 Remove the primary antibody, and wash the membrane with TBS-T at room temp for 10 min. Repeat this procedure three times.

2.3.4 Add secondary antibody diluted in blocking solution. Incubate at room temperature for 1 h with gentle shaking.

2.3.5 Remove the secondary antibody.

2.3.6 Wash the membrane with TBS-T at room temperature for 10 min. Repeat this procedure six to eight times.

2.3.7 After the final wash, drain TBS-T as much as possible and add a chemiluminescent reagent.
